# Toll Like Receptor 3 Plays a Critical Role in the Progression and Severity of Acetaminophen-Induced Hepatotoxicity

**DOI:** 10.1371/journal.pone.0065899

**Published:** 2013-06-07

**Authors:** Karen A. Cavassani, Ana Paula Moreira, David Habiel, Toshihiro Ito, Ana Lucia Coelho, Ron M. Allen, Bin Hu, Janna Raphelson, William F. Carson, Matthew A. Schaller, Nicholas W. Lukacs, M. Bishr Omary, Cory M. Hogaboam, Steven L. Kunkel

**Affiliations:** 1 Department of Pathology, University of Michigan, Ann Arbor, Michigan, United States of America; 2 Department of Internal Medicine, University of Michigan, Ann Arbor, Michigan, United States of America; 3 Department of Molecular and Integrative Physiology, University of Michigan, Ann Arbor, Michigan, United States of America; Centre d'Immunologie de Marseille-Luminy, CNRS-Inserm, France

## Abstract

Toll-like receptor (TLR) activation has been implicated in acetaminophen (APAP)-induced hepatotoxicity. Herein, we hypothesize that TLR3 activation significantly contributed to APAP-induced liver injury. In fasted wildtype (WT) mice, APAP caused significant cellular necrosis, edema, and inflammation in the liver, and the *de novo* expression and activation of TLR3 was found to be necessary for APAP-induced liver failure. Specifically, liver tissues from similarly fasted TLR3-deficient (*tlr3^−/−^*) mice exhibited significantly less histological and biochemical evidence of injury after APAP challenge. Similar protective effects were observed in WT mice in which TLR3 was targeted through immunoneutralization at 3 h post-APAP challenge. Among three important death ligands (i.e. TNFα, TRAIL, and FASL) known to promote hepatocyte death after APAP challenge, TNFα was the only ligand that was significantly reduced in APAP-challenged *tlr3^−/−^* mice compared with APAP-challenged WT controls. *In vivo* studies demonstrated that TLR3 activation contributed to TNFα production in the liver presumably via F4/80^+^ and CD11c^+^ immune cells. *In vitro* studies indicated that there was cooperation between TNFα and TLR3 in the activation of JNK signaling in isolated and cultured liver epithelial cells (i.e. nMuLi). Moreover, TLR3 activation enhanced the expression of phosphorylated JNK in APAP injured livers. Thus, the current study demonstrates that TLR3 activation contributes to APAP-induced hepatotoxicity.

## Introduction

Acetaminophen (N-acetyl-para-aminophenol (APAP)) overdose remains one of the most common reasons for drug-induced liver injury in the United States and the United Kingdom, accounting for approximately one third of the cases of acute liver failure [1]. While it has been recognized that APAP-induced acute liver failure is a preventable cause of death, it continues to be a growing and significant public health problem [2], [3]. APAP-induced hepatotoxicity is the consequence of the generation of toxic metabolites from APAP, which lead to hepatocyte death by necrosis and apoptosis. Hepatocyte death leads to secondary activation of the innate immune response involving upregulation of inflammatory cytokines and chemokines and the infiltration of various inflammatory cell types [4]–[6]. The mechanism(s) leading to the initial hepatocyte injury and subsequent inflammatory response during APAP-induced acute liver failure has generated considerable research interest since a more complete understanding of this process might lead to viable therapeutic options following APAP overdose.

Toll-like receptors (TLR) are important receptors in the recognition of pathogen-associated molecular patterns (PAMPs) during infection. However, it is also apparent that regardless of their cellular localization, this family of receptors can recognize endogenous ligands released from dying cells during tissue injury [7]. Because TLRs respond to these endogenous ligands, there is a growing awareness that TLR-driven innate immune responses might precipitate severe pathophysiologic consequences even in the absence of infectious agents. APAP-induced hepatotoxicity promotes the release of mitochondrial DNA leading to TLR9 receptor activation [8], [9]. Likewise TLR3 has been shown to respond to endogenous RNA released from dying cells during injury to the joint [10], gut [11], skin [12], [13], or central nervous system [14]. While the signaling mechanisms propagated following TLR3 engagement of viral dsRNA or the synthetic dsRNA analog PolyI:C have been described in the liver [15], [16], the signaling mechanism(s) evoked by endogenous factors binding to TLR3 during acute hepatotoxicity are less well understood.

TNFα is generated during APAP-mediated hepatotoxicity and has a dual role in the liver depending on its level of expression and the presence of other inflammatory signals [17]. Overexpression of TNFα can lead to liver injury and failure of liver regeneration. Under certain circumstances including overexpression, TNFα promotes JNK activation [18]. In fact, the cytoprotective effects of NF-κB activation during liver injury appear to be mediated, in part, through its suppression of the JNK pathway [19]. Studies involving either the inhibition of JNK via pharmacological compounds or gene silencing with antisense oligonucleotides have clearly demonstrated that the JNK pathway contributes to APAP-induced liver hepatotoxicity [20], [21].

Given that TLR3 is activated during non-infectious tissue damage, we examined the manner in which TLR3 activation contributes to APAP-induced liver injury. The present study demonstrates that TLR3 activation is required for APAP-induced hepatotoxicity. These results were confirmed via the administration of a neutralizing Abs directed against mouse TLR3, which provided a significant protective effect in wild-type (i.e. *tlr3^+/+^*) mice. Moreover, TLR3 activation was found to regulate the inflammatory response, most notably the TNFα pathway following APAP challenge in mice. *In vitro* studies suggested that there was cooperation between TNFα and TLR3 agonists that leads to hepatocyte death. Together, these results demonstrate that TLR3 activation contributes to APAP-induced hepatotoxicity.

## Materials and Methods

### Mice

Specific pathogen-free, female C57BL/6 (wildtype; WT) mice (6 to 8 weeks; Taconic Company, Germantown, NY) were housed at the University of Michigan. *Tlr3* ^−/−^ mice were originally provided by Dr. Richard Flavell (Yale School of Medicine, New Haven, CT); a breeding colony of *Tlr3^−/−^* mice was established at the University of Michigan. Dr. Mark Kaplan (Indiana University, Indianapolis, IN) provided the mice lacking the *Ifn*a*R* gene (*IFN*α*R^−/−^*) used in the present study. All knockout strains of mice were backcrossed a minimum of 10 generations onto a C57BL/6 background. Age- and sex-matched mice were used for all of the studies described herein. The University Committee on the Use and Care of Animals at the University of Michigan approved all *in vivo* protocols used in this study.

### APAP-induced hepatotoxicity

APAP (Sigma-Aldrich, St. Louis, MO) solution was made fresh for each experiment in PBS (pH = 7.4) at 10 mg/ml and heated in a water bath to 56°C to dissolve. APAP was dosed at 300 mg/Kg via an i.p. injection into mice fasted for 14–16 h, as previously described in detail [22]. Mice were euthanized by ketamine/xylazine injection prior to the collection of serum and liver tissues for mRNA, protein, histologic, western blotting, and immunofluorescence analysis at indicated time points. Untreated mice at the 0 h timepoint correspond to both WT and *tlr3* ^−/−^ mice that were fasted for 16 h but not challenged with APAP. In other experiments, WT mice received one of the following treatments: 1) control rabbit IgG (2 mg/mouse); 2) rabbit anti-TLR3 polyclonal antibody (2 mg/mouse; [11]) at 3 h after APAP; 3) control rat IgG mAb (500 µg/mouse; Jackson Laboratories); or (4) mouse anti-TLR3 monoclonal antibody (500 µg/mouse; Centocor, Radnor, PA) at 3 h after APAP. At 24 h after the APAP challenge, serum and liver were removed for biochemistry and/or histological analysis. A kit from Dojindo Molecular Technologies was used to measure glutathione (GSH) in WT and *tlr3* ^−/−^ fasted livers according to provided instructions. Serum samples were taken from fasted WT and *tlr3* ^−/−^ mice at 0.5, 1, 2, 4, and 8 h after APAP (300 mg/Kg) to measure APAP concentrations using the Acetaminophen Direct ELISA Kit (Immunalysis) according to the manufacturer's instructions.

### Histological and Immunofluorescence analysis

For histological analysis, excised liver and lung samples were fixed with 10% buffered formalin and processed using routine histological techniques. Tissue sections were stained with H/E or PAS (to identify glycogen storage) and analyzed by light microscopy. For immunofluorescence analysis, livers were embedded in Tissue-Tek OCT compound (Sakura Finetek, Torrance, CA) and snap frozen in liquid nitrogen. Seven-micron cryostat sections were fixed in ice-cold acetone. Fc receptors were blocked with a 2.4G2 mAb for 30 min at room temperature. Each slide was then stained with a primary antibody overnight at 4°C followed by a secondary antibody stain for 1 h at room temperature. Washes between antibody incubations were performed using PBS+0.05% Tween 20. Coverslips were mounted on each slide using ProLong® Gold Antifade Reagent with DAPI (Invitrogen). The following primary antibodies were used: rat anti-mouse keratin 8; rabbit anti-mouse TLR3 [11]; biotin-labeled anti-mouse F4/80 (Serotec); biotin-labeled anti-mouse CD11c (Biolegend). An appropriate Alexa-labeled secondary reagent (Invitrogen) followed these primary mAb. The stained tissue sections were analyzed using a Nikon A1 confocal microscope system (Nikon Instruments) and Olympus BX43 Fluorescence microscope (Cells Sens Software).

### Reverse Transcription and Real-time Quantitative PCR Analysis

Total RNA was isolated from whole livers using TRIzol (Invitrogen) or from cultured cells using a RNAeasy Micro Kit (Qiagen) according to the manufacturer's instructions. Briefly, a total of 2.5 µg (whole livers) or 100–500 ng (cells) of RNA was reverse transcribed to yield cDNA in a 25-µl reaction mixture containing 1× first strand (Life Technologies; Invitrogen), 250 ng oligo (dT) primer, 1.6 mmol/l dNTPs (Invitrogen), 5 U RNase inhibitor (Invitrogen), and 100 U of Moloney murine leukemia virus reverse transcriptase (Invitrogen) at 38°C for 60 min. The reaction was stopped by incubating the cDNA at 94°C for 10 min. An ABI 7700 Sequence Detector System (PE Applied Biosystems) was used to perform real-time quantitative PCR analysis. Thermal cycling was performed at 50°C for 2 min and 95°C for 10 min, followed by 40 cycles of amplification at 95°C for 15 s and 55°C for 1.5 min for denaturing and annealing, respectively. The mRNA expression was normalized to *Gapdh*, and the fold change in transcript expression was calculated between experimental groups as indicated in the graphs. For tissue analysis, all fold changes were calculated via a comparison to mRNA expression in naïve liver while for cell analysis, all fold changes were calculated via a comparison to mRNA expression in cells exposed to growth media alone. All primers were purchased from Applied Biosystems (Tlr3, Mm01207404_m1*; Ccl2, Mm00441242_m1*; Ccl3, Mm00441259_g1*; Cxcl1 (KC), Mm04207460_m1*; Cxcl10, Mm00445235_m1*).

### Protein analysis

Before each assay, snap-frozen liver samples were thawed on ice, and homogenized in a solution containing 2 mg of protease inhibitor (Complete; Boehringer Mannheim, Indianapolis, IN) per milliliter of normal saline. Organ extracts were centrifuged (12000× *g* for 10 min at 4°C) to remove all particulate material. Murine cytokines levels were measured using a Bio-Plex (Bio-Rad Laboratories) or ELISA (R&D Systems) assay. The cytokine levels in liver homogenates were normalized to the protein levels measured using a Bradford assay. Alanine aminotransferase (ALT) and aspartate aminotransferase (AST) were measured in serum samples using standardized clinical assays by ULAM PCAR Animal Diagnostic Laboratory from University of Michigan.

### Culture of liver epithelial cell lines

Normal murine liver cells (ATCC CRL-1638; NmuLi) were cultured in FBS-deficient DMEM supplemented with antibiotics for 36 h prior to an *in vitro* experiment. Following this ‘fasting’ phase, the cells were cultured with one or more of the following: 10 mM of APAP, 20 ng/ml TNFα, 10 µg/ml RNA from necrotic nMuLi, and/or 10 µg/ml PolyI:C. Necrotic RNA was isolated from fasted (20 h) nMuLi cells exposed to 20 mM of APAP. After 8 h of incubation, the cells were centrifuged at 4000 rpm for 5 min and the mRNA was isolated by Trizol method.

### Isolation of primary hepatocytes from APAP-challenged livers

Primary hepatocyte isolation was performed by collagenase perfusion as previously described in detail by Hu *et al* [23] with few modifications. Briefly, general anesthesia was induced with 50 mg/Kg of pentobarbital, midline laparotomy performed, and the inferior vena cava was exposed and cannulated with a 26-gauge angiocatheter. The liver was perfused with warm liver perfusion buffer (GIBCO) at a rate of 3 ml/min for 5 min to flush the liver of intravascular blood. Next, warm liver digest buffer (dPBS supplemented with 1.0M CaCl2, 1.0M Glucose, penicillin-streptomycin, and approximately 3600 U Collagenase IV (Worthington)) was infused at a rate of 3 ml/min for 7 min to digest the liver. The isolated liver was placed a petri dish, minced into 1-mm pieces, and gently agitated to disperse the cells in William's E medium (Gibco). The cell suspension was then filtered through 100 micron cell strainer (BD falcon), washed twice at 500 rpm at 4°C for 2 min, and the hepatocytes were plated on collagen I-coated plates. After 3 h of incubation with incomplete William's E medium (Gibco), hepatocytes were washed and the adherent cells were cultured with William's Medium E supplemented with 10% FCS, 10,000 U/ml penicillin-streptomycin, 2 mM L-glutamine, 10 mM HEPES, 132 ng/ml dexamethasone at 37°C under 5% CO_2_. The isolated hepatocytes were stimulated with one or more of the following: 5 mM of APAP, 20 ng/ml TNFα (R&D Systems), 10 µg/ml PolyI:C (InvivoGen),10 µg/ml necrotic RNA (as described above). RT-PCR was used to analyze the expression of TLR3 at 24 h post-stimulus. The viability of hepatocytes following stimulus was determined using Presto Blue® Cell Viability Reagent (Invitrogen). Fluorescence was analyzed using an EnSpire 2300 Multilabel Reader via Enspire Manager Software.

### Flow Cytometry

Analysis of liver DCs, liver Kupffer cells, and liver sinusoidal endothelial cells (LSECs) was performed according to Zellweger *et al* [24]. The livers from WT and *tlr3* ^−/−^ mice were collected 24 h post-APAP, and minced and digested for 20 min at 37°C with agitation in complete RPMI containing 10% fetal calf serum, Penicillin-Streptomycin, L-glutamine and 1.5 mg/ml of collagenase VIII (Sigma) in approximately 15 ml. Tissues were immediately washed in complete RPMI at 1500 rpm for 5 min at room temperature. The pellets were re-suspended and pressed through 100 µm cell strainers (BD) using the plunger of a 3 ml syringe (BD). The cells were collected and pelleted by centrifugation (1500 rpm 5 minutes at RT) and re-suspended in a 14.7% Optiprep (Sigma) solution diluted in 150 mM NaCl with 10 mM HEPES (pH = 7.4) and layered over 22.2% of Optiprep solution diluted in the same buffer. Cells were spun down for 20 min at 700× g at 25°C (acceleration 6, deceleration 1). The interphase was collected and washed immediately after with RPMI. FcγRs were blocked with mAb 2.4G2 and the cells were stained for surface markers using anti-CD11c (eBioscience), anti-Gr1, anti-F4/80, anti-CD45, anti-CD31 (from Biolegend) and then resuspended in fixation/permeabilization solution (BD Cytofix/Cytoperm Kit; BD Biosciences-Pharmingen). Rat IgG1, kappa light chain was used as isotype control for TNFα staining. Data were collected using an LSR II Flow cytometry (BD) and analyzed with FlowJo software (Tree Star).

### Western Blot analysis

Cells and liver tissues were lysed and centrifuged at 15,000× *g* for 10 min. Equal amounts of cell lysates (20–50 µg) were fractionated by SDS-PAGE (NuPage; Invitrogen) and subsequently transferred onto nitrocellulose (Invitrogen). After an overnight incubation with the appropriate primary mAbs for p-JNK/SAP, JNK/SAP (Cell Signaling Technology) or a pAb for TLR3 [11] (dilution 1∶1000), the membranes were counterstained with peroxidase-conjugated anti-rabbit IgG and visualized with enhanced chemiluminescence detection reagents (ECL; GE Healthcare). Membranes were stripped in Restore™ Buffer (Pierce) at 50°C for 45 min, blocked with 5% non-fat milk, and re-probed with anti-GAPDH (Santa Cruz) or ß-actin (Sigma) followed by peroxidase-conjugated anti-mouse IgG. The images were analyzed using Image J 1.42q (freeware available from the National Institutes of Health).

### Statistical analysis

Statistical differences were detected using the unpaired Student's *t*-test. Values are presented as mean ± SEM. *P* values ≤0.05 were considered statistically significant. Calculations were performed using the Prism 5.0 software program for Apple Computers (GraphPad Software, San Diego, CA)

## Results

### TLR3 expression in the APAP-damaged liver

**Figure 1:**
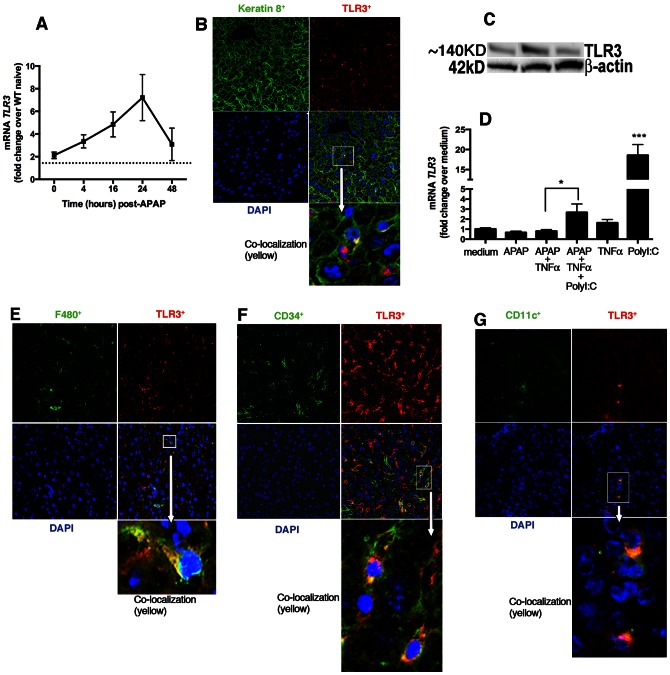
TLR3 expression in liver after APAP challenge. (**A**) Quantitative real-time PCR (TaqMan) was performed to measure the transcript levels of TLR3 in whole liver homogenates at 0, 4, 16, 24 and 48 h after the injection of APAP. Data shown indicate mean ± SEM of two independent experiments (*n* = 9–10 per time point). (**B**) Confocal immunofluorescent examination of livers revealed TLR3+ cells (red) and the keratin 8+ cells (green) surrounding the areas of necrosis in WT mice at 24 h after APAP injection. Shown are representative sections from 1 mouse of 5 per group. (**C**) The expression of TLR3 was analyzed in primary hepatocytes by western blotting. Each lane represents isolated hepatocytes from independent experiments. (**D**) Expression of TLR3 transcript levels in primary hepatocytes. 1.5×10^5^ cells/well were plated in 24 well-plates and stimulated with the indicated stimulus (5 mM of APAP, 20 ng/ml of TNFα, 10 µg/ml of PolyI:C) for 24 h. Bars represents mean±SEM of three independent experiments. *p<0.05 when APAP+TNFα+PolyI:C was compared with APAP+TNFα; ***p<0.0001 when only PolyI:C was compared with medium. Immunofluorescence analysis of livers revealed TLR3+ cells (red) in (**E**) Kupffer cells (F4/80), (**F**) CD34, and (**G**) CD11c in liver sections from WT mice at 24 h after APAP. Magnification: 400×. Shown are representative sections from 1 mouse of 5 per group.

Previous studies have shown that TLRs are expressed in the liver under homeostatic conditions but at lower levels than in other organs in the gastrointestinal tract [25]. The expression of TLR2, TLR4 and TLR3 in hepatocytes is upregulated under inflammatory conditions [26], [27]. To examine whether TLR3 expression in whole liver was altered during APAP-induced hepatotoxicity, quantitative RT-PCR was first employed to determine temporal changes in TLR3 expression in the naïve and APAP-challenged liver. The expression of TLR3 mRNA peaked in WT mice at 24 h after APAP and remained elevated at 48 h after APAP (**Figure 1A**). Since centrilobular veins are the areas in the liver that are most susceptible to APAP, all image analysis focused on this area of the liver. It was apparent using immunofluorescence techniques that WT livers contained higher amounts of TLR3 protein after APAP challenge (unchallenged livers are shown in **Figure S1A** whereas APAP-challenged livers at 24 h are shown in **Figure 1B**). It was also apparent that TLR3 (indicated by red staining) was expressed in hepatocytes (i.e. the Keratin 8^+^ positive cells highlighted in green staining) and also in other cells localized in the liver during APAP injury (**Figure 1B**). No expression of TLR3 was detected in livers from *tlr3^−/−^* mice after APAP challenge (**Figure S1B**), confirming the specificity of this polyclonal Ab [11]. TLR3 protein was also examined in naive primary hepatocytes using a Western blotting technique (**Figure 1C**; *n* = 3 separate primary cells). To examine the regulation of TLR3 mRNA in primary hepatocytes, these cells were exposed to various stimuli for 24 h prior to quantitative PCR analysis. Although TLR3 expression in primary hepatocytes was not affected by APAP challenge alone, the addition of TNFα+a TLR3 ligand (PolyI:C) or PolyI:C alone significantly increased the expression of this receptor (**Figure 1D**). Finally, immunofluorescence analysis demonstrated that F4/80 (**Figure 1E**), CD34 (**Figure 1F**), and CD11c (**Figure 1G**) all co-localized with TLR3 in APAP-challenged WT livers suggesting that Kupffer cells (F4/80+), Liver sinusoidal endothelial cells (LSECs), and CD11c+ (possibly macrophages or DCs) express TLR3 following APAP-induced hepatotoxicity. Taken together, these data indicated that TLR3 was constitutively expressed in naïve liver but its expression was markedly induced in hepatocytes and immune cells following injury induced by APAP challenge.

### TLR3 activation contributes to APAP-induced hepatotoxicity

**Figure 2:**
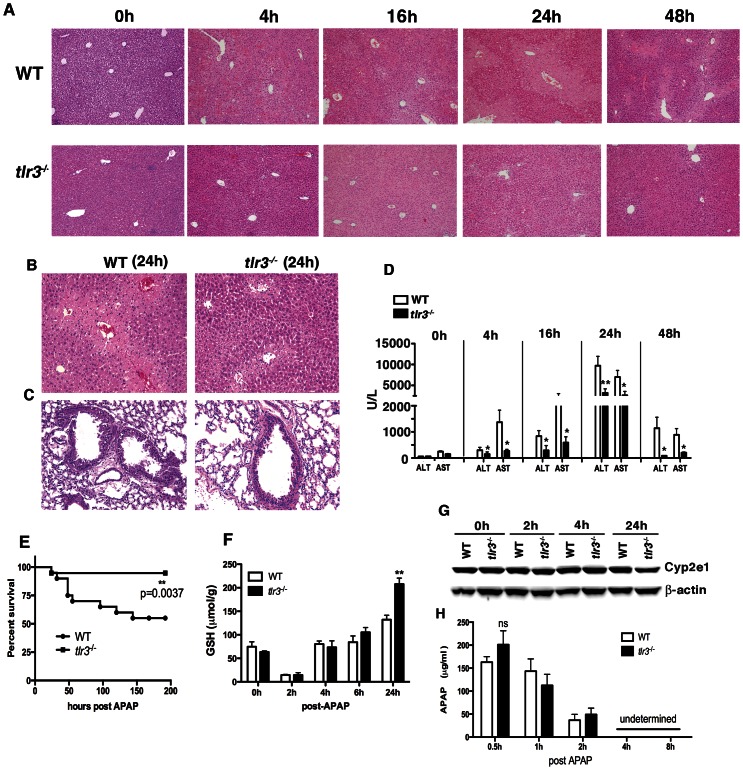
*Tlr3* ^−/−^ mice exhibit markedly less hepatotoxicity following APAP. (**A–B**) Representative histological liver samples from WT and *tlr3* ^−/−^ mice after APAP. (**C**) Lung sections from WT mice at 24 h post APAP. Original magnifications: (**A**) 100× or (**B–C**) 200×. (**A–C**). Data from 4 and 16 h after APAP are representative of 2 independent experiments (*n* = 5 mice/group). Data from 24 h after APAP are representative of 5 independent experiments (*n* = 5 mice/group), and data from 48 h post APAP is representative of 3 independent experiments (n = 4–9 mice/group). (**D**) Serum ALT and AST levels were measured at various time points after APAP. Values represent mean±SEM obtained from 2–3 independent experiments (n = 4–5 per group) (*p<0.05; **p<0.01). (**E**) Survival of WT and *tlr3* ^−/−^ mice after 500 mg/kg of APAP. The data are from 2 independent experiments. Each experiment was performed with 10 WT mice and 9–10 *tlr3* ^−/−^ mice. **p = 0.0037 (Mantel-Cox test). (**F**) Hepatic GSH levels after APAP injection (*n* = 3–4 mice/group). Values represent means±SEM of 2 independent experiments. **p<0.01 when *tlr3* ^−/−^ was compared with WT mice. (**G**) Liver lysates of WT and *tlr3* ^−/−^ mice were prepared and pooled for Western blotting analysis using anti-Cyp2e1 and β-actin antibodies. (**H**) Concentration of APAP was determined in serum from WT and *tlr3* ^−/−^ at the indicated time points. Values indicate mean ± SEM of 5–6 individual mice. ns = not significant.

Liver histology was assessed at 0, 4, 16, 24, and 48 h after APAP injection in WT and *tlr3* ^−/−^ mice. APAP caused tissue necrosis in the centrilobular regions of WT mice (**Figure 2A**). In contrast, the same dose of APAP caused little histological injury in fasted *tlr3* ^−/−^ mice (**Figure 2A**) suggesting that the absence of TLR3 activation inhibit the progression of liver injury. In fact, at 24 h, less tissue injury was apparent in liver samples from *tlr3* ^−/−^ mice compared with liver from WT mice (**Figure 2B**). APAP-induced lung injury was also ablated in the *tlr3* ^−/−^ group compared with the WT group (**Figure 2C**). Quantitative evidence of the tissue protective effect provided by the absence of TLR3 is shown in **Figure 2D**. Compared with WT mice, *tlr3* ^−/−^ mice showed significantly lower serum levels of ALT and AST at 4, 16, 24, and 48 h after APAP. Importantly, at 48 h the levels of AST and ALT in *tlr3* ^−/−^ mice decreased to near basal levels (**Figure 2D**). APAP-induced mortality due to a single dose at 500 mg/kg was significantly decreased in *tlr3* ^−/−^ mice (**Figure 2E**). Finally, WT and *tlr3^−/−^* mice exhibited a similar loss in GSH levels at 2 h after APAP but levels of this factor were significantly higher at 24 h after APAP in the knockout group compared with the WT group (**Figure 2F**), which reflects low APAP hepatotoxicity in *tlr3^−/−^* mice. No significant differences were found in the liver levels of Cyp2e1 between WT and *tlr3^−/−^* mice prior to and after APAP (**Figure 2G**). In addition, no significant differences in APAP serum levels between WT and *tlr3* ^−/−^ mice were observed, indicating that both groups were able to metabolize APAP to the same extent (**Figure 2H**). Although the genetic background of mice can affect APAP-induced hepatotoxicity [28], we did not find any differences in the levels of ALT and AST after APAP in WT mice purchased from either Taconic or Jackson Laboratories (**Figure S2**). This was an important finding in light of the fact that the *tlr3^−/−^* mice were fully backcrossed using a Jackson C57BL/6 mouse whereas the WT C57BL/6 controls we employed in these studies were from Taconic. Together, these findings showed that genetic deletion of TLR3 in mice significantly attenuated APAP-induced hepatotoxicity and lethality independently of any discernable effects on the metabolism of this compound.

Since we observed that TLR3 was expressed in hematopoietic and non-hematopoietic cells in the liver after APAP, we next examined the response of bone marrow (BM) chimeric mice during APAP-induced hepatotoxicity. WT BM transfer to lethally-irradiated WT mice (WT→WT) expressed the highest levels of AST and ALT, and the greatest histological injury compared with the other bone marrow transfer conditions examined including *Tlr3* ^−/−^→WT, *tlr3* ^−/−^→*tlr3* ^−/−^, or WT→*tlr3* ^−/−^ BM chimeric mice (**Figure S3**). Thus, these data demonstrated that TLR3 activation in both BM derived and liver resident cells contributed to the liver damage following APAP challenge.

### Antibody-mediated immunoneutralization of TLR3 attenuates the APAP-induced hepatotoxicity in wildtype mice

**Figure 3:**
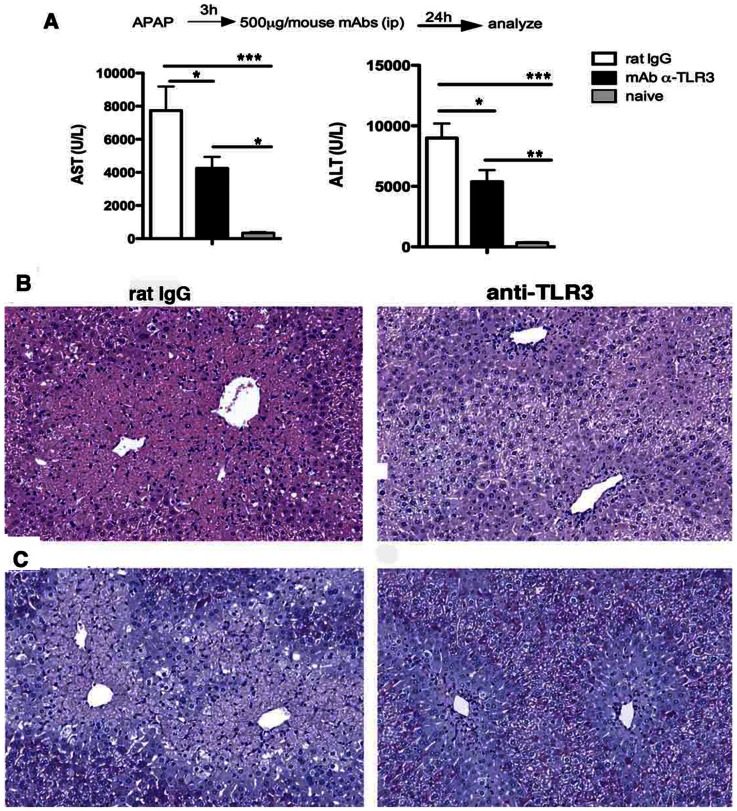
Anti-TLR3 mAb markedly reduced liver injury induced by APAP challenge. Groups of fasted WT mice received APAP (300 mg/kg; i.p. injection), and either rat IgG or anti-TLR3 mAb antibody. Both groups were analyzed at 24 h after APAP injection. (**A**) AST and ALT serum levels from WT mice that received anti-TLR3 mAb 3 h after the APAP injection; enzymes levels from naïve mice were also measured. (**B**) Representative liver sections stained with H&E or (**C**) PAS from WT mice that received either IgG or anti-TLR3 mAb at 3 h after APAP challenge. All tissues were removed at 24 h after APAP challenge. Magnification, 200×. Data in all panels are representative of two independent experiments; *n* = 5 mice per group.

Given that the genetic absence of TLR3 provided a protective effect during APAP-induced hepatotoxicity, the next series of experiments addressed whether this effect could be achieved through antibody-mediated immunoneutralization of TLR3. APAP-challenged mice that received the monoclonal anti-TLR3 antibody at 3 h after APAP challenge exhibited significantly lower levels of AST and ALT (**Figure 3A**) and significantly less liver injury at 24 h as determined by H&E staining (**Figure 3B**) and PAS staining (**Figure 3C**). Similarly, the administration of a polyclonal anti-TLR3 antibody at 3 h after APAP challenge significantly protected WT mice from APAP-induced hepatotoxicity (**Figure S4)**. Unlike the IgG control group, treatment with anti-TLR3 antibody resulted in significantly lower serum ALT and AST levels (**Figure S4A**). Further, smaller areas of tissue necrosis surrounding the centrilobular veins were found in the anti-TLR3 treated mice and the PAS staining showed an increased area of glycogen storage by hepatocytes, which is inversely related to damage to the hepatocytes (**Figure S4B and S4C**). Also, anti-TLR3 antibody administration resulted in reduced damage in the lung epithelial cells when compared with the administration of rabbit IgG (**Figure S4D**). Together, these data demonstrate that the beneficial effects of *tlr3* gene deficiency are also achieved via an anti-TLR3 antibody directed approach.

### TLR3 is necessary for the inflammatory response in the liver following APAP challenge

**Figure 4:**
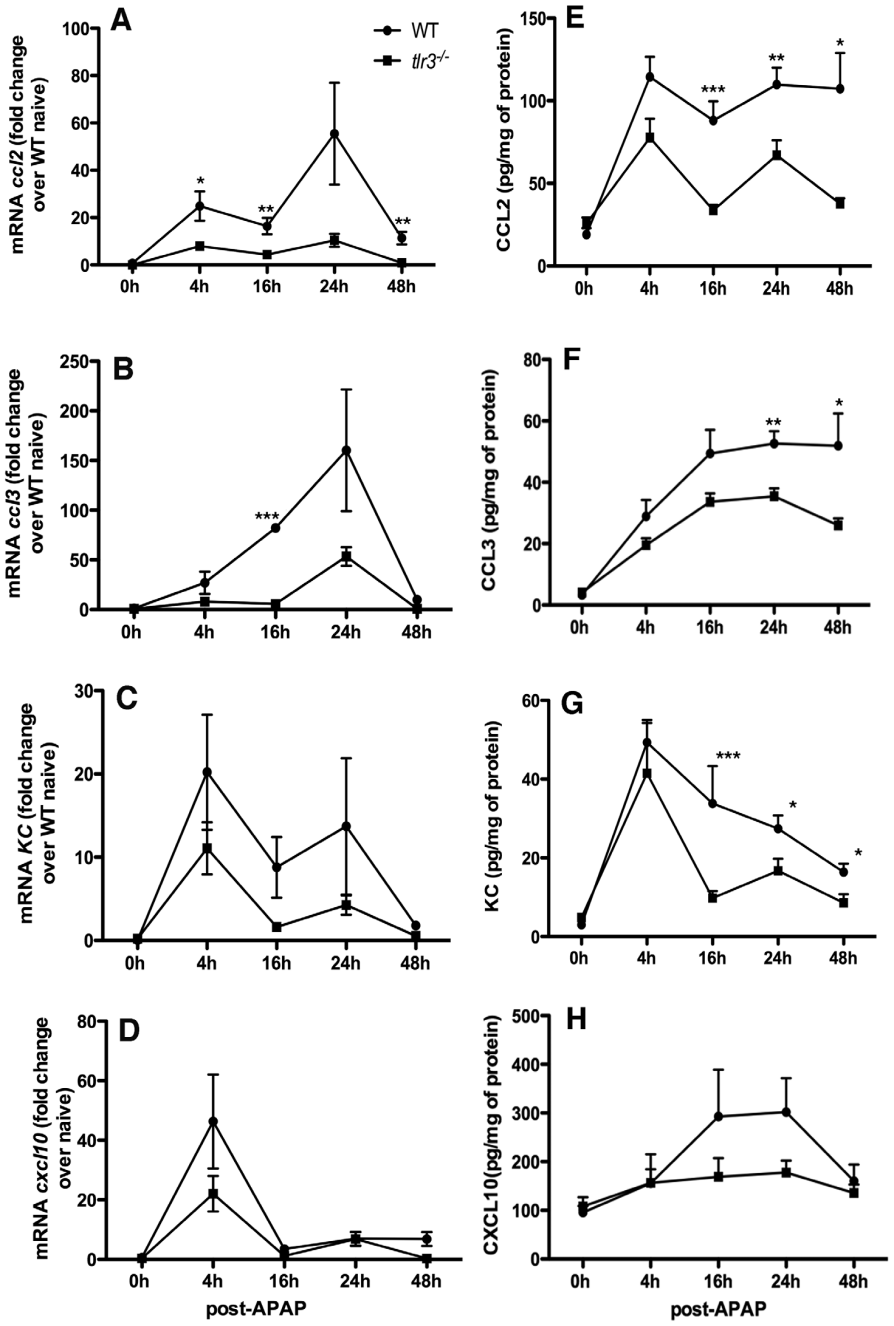
TLR3 activation increased chemokine levels following APAP-induced hepatotoxicity. Chemokine transcript levels (**A–D**) and protein levels (**E–H**) in WT and *tlr3* ^−/−^ livers homogenates after APAP. The data represent mean±SEM obtained from 2–3 independent experiments (*n* = 5/group). *p<0.05; **p<0.01 and ***p<0.001 when WT were compared with *tlr3* ^−/−^ mice.

The generation of chemokines in liver from WT and *tlr3* ^−/−^ mice was determined following APAP challenge. Liver mRNA and protein levels for CCL2, CCL3, KC, and CXCL10 at 0, 4, 16, 24, and 48 h post APAP challenge were measured (**Figure 4A–D**). The expression of CC chemokines was significantly increased and reached peak expression at 24 h after administration of APAP in livers from WT but not from *tlr3* ^−/−^ mice (**Figure 4A–B**). In APAP challenged mice, CCL2 and CCL3 protein levels were increased in WT mice but not in *tlr3* ^−/−^ mice at all time points analyzed (**Figure 4E–F** **)**. No significant difference was detected in *KC* mRNA expression between the WT and *tlr3* ^−/−^ groups (**Figure 4C**). The expression of *Cxcl10* mRNA in WT livers was similar to those measured in *tlr3* ^−/−^ livers (**Figure 4D**). Unlike the mRNA expression, KC protein levels were increased in WT livers, with the peak at 16 h after APAP (**Figure 4G**). The protein levels of CXCL10 were not significantly different between WT and *tlr3* ^−/−^ at any time point analyzed (**Figure 4H** **)**. The levels of the chemokines CCL2, CCL3, KC, and CXCL10 in livers were significantly reduced compared with the control group when the anti-TLR3 pAb was administered after APAP challenge (**Figure S5**). Collectively, APAP challenge in *tlr3* ^−/−^ but not in WT mice was associated with a marked decrease in hepatic chemokine generation indicating a reduced liver injury.

IFNα is a major cytokine product of TLR3 activation leading us to examine whether this cytokine contributed to APAP-induced hepatotoxicity. First, we observed a significant decrease in the levels of IFNα4 in total livers from *tlr3* ^−/−^ mice compared with WT after APAP (**Figure S6A**). However, mice lacking the IFNα receptor (*ifnαR* ^−/−^) exhibited similar liver TNFα levels (**Figure S6B**), liver chemokine levels (**Figure S6C**), serum AST and ALT levels (**Figure S6D**), and liver injury (**Figure S6E–F**) compared with the appropriate WT mice at 24 h after APAP challenge. Together, these findings demonstrate that type I interferons are not major contributors to APAP-induced hepatotoxicity.

### TLR3 activation regulates the expression of death ligands during APAP-induced hepatotoxicity

**Figure 5:**
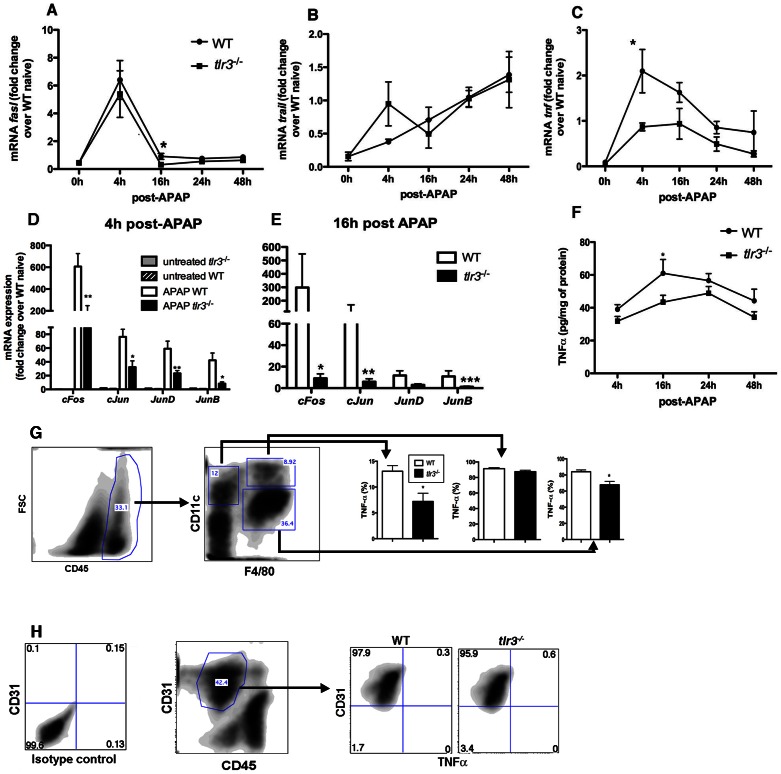
TLR3 modulates the expression TNFα after APAP. Total liver transcript levels of (**A**) *fasl*, (**B**) *trail*, (**C**) *tnf* and (**D–E**) AP-1 genes in WT and *tlr3* ^−/−^ mice. (**F**) Total liver TNFα protein levels in WT and *tlr3* ^−/−^ mice after APAP. Statistically significant differences are indicated (*p<0.05; **p<0.01 and ***p<0.001). Data are presented as the mean ± SEM of 2–3 independent experiments. (**G–H**) Percentage of TNFα+ cells in different liver cell populations isolated from WT and *tlr3^−/−^* mice at 24 h post APAP. Gating is indicated on the left, population statistics are indicated in the bar graph on the right. Rat IgG1, kappa light chain antibody was used as an isotype control for TNFα staining (**Figure S7**). Representative population of LSECs (CD45^−^CD31^+^) is shown. Data is representative of two independent experiments. Data represent mean ± SEM (*n* = 3/group in each experiment).

To better understand the mechanisms through which TLR3 contributed to liver injury after APAP challenge, we next analyzed the expression of *fasl*, TNFα-related apoptosis inducing ligand (*trail*) and *tnfα* at the indicated time points after APAP-induced injury in the livers from WT and *tlr3* ^−/−^ mice. The expression of *fasl* peaked at 4 h post APAP, however no major differences were found between WT and *tlr3* ^−/−^ mice. (**Figure 5A**). *Trail* expression was not modulated following APAP challenge, and no difference was observed between WT and *tlr3* ^−/−^ mice (**Figure 5B**). Finally, the expression of *tnf* was significantly increased in response to APAP in WT mice but not in *tlr3* ^−/−^ mice (**Figure 5C**).

The JNK/AP-1 signaling pathway is activated during a hepatotoxic response in the liver [29], [30]. This and the lower expression of *tnf* suggested to us that the *tlr3* ^−/−^ liver might exhibit profound defects in AP-1-dependent gene expression during APAP challenge. Indeed, the expression of the AP-1-target genes TNFα, *cFos*, *cJun*, *JunD*, and *JunB* were reduced in the liver of APAP-treated *tlr3* ^−/−^ mice compared with APAP-treated WT mice (**Figure 5D–E**).

Under certain conditions, TNFα has been shown to promote JNK activation and hepatocyte death [30], findings that led us to further characterize the expression and functions of this cytokine during APAP-induced hepatotoxicity. Our first observation was that TNFα protein levels in WT livers were significantly increased at 16 h but only modestly increased at all other times after APAP compared with *tlr3* ^−/−^ livers (**Figure 5F**). To investigate the identity of the cells that were expressing TNFα in WT and *tlr3* ^−/−^ mice after APAP, total mononuclear cells were purified from whole liver, stained for surface molecules and intracellular TNFα, and analyzed by flow cytometry. Cells were gated according to the expression of CD45 and in this gate we identified three distinct cell populations that expressed TNFα: 1) CD11c^+^F4/80^−^TNFα^+^; 2) CD11c^+^F4/80^+^TNFα^+^; 3) CD11c^−^F4/80^+^TNFα^+^. In *Tlr3* ^−/−^ mice, the percentage of TNFα^+^CD11c^+^F4/80^−^ and TNFα^+^CD11c^−^F4/80^+^ cells was significantly lower than was found in the livers of WT mice (**Figure 5G**). Although cells expressing both CD11c and F4/80 receptors were found to be positive for TNFα, we did not find any significant differences in their numbers between WT and *tlr3* ^−/−^ mice after APAP (**Figure 5G**). LSECs (CD45^−^CD31^+^) were found to express TLR3 in WT mice after APAP (**Figure 1F**) but these cells were not positive for TNFα (**Figure 5H**). Gr1^+^ cells were also negative for TNFα (data not shown). Finally, analysis of TNFα in supernatants taken from primary hepatocytes cultures indicated that these cells were not a source of TNFα (data not shown). Together, these data suggest that the increased TNFα expression in WT versus *tlr3^−/−^* mice after APAP might be due to altered TNFα generation by CD11c^+^F4/80^−^ (possibly DCs) and CD11c^−^F4/80^+^ cells (possibly Kupffer cells).

**Figure 6:**
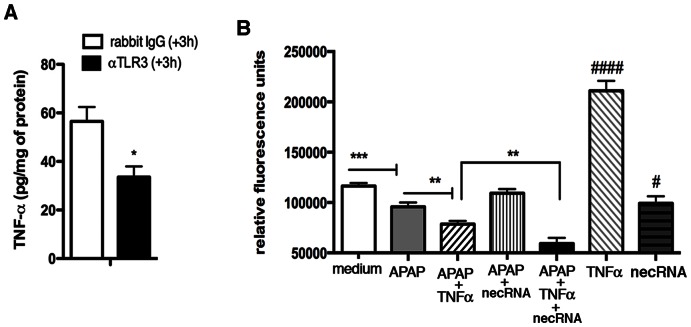
TLR3 activation leads to TNFα generation, which synergizes with TLR3-mediated effects in hepatocytes. (**A**) TNFα protein levels were measured in fasted WT mice that received either control rabbit IgG or anti-TLR3 mAb at 3 h after APAP injection (300 mg/kg; i.p. injection). The data represent mean ± SEM from 2 independent experiments (*n* = 5 per group/experiment). *p<0.05; when IgG treated group was compared with anti-TLR3 antibody treatment. (**B**) 2.0×10^4^ mouse WT primary hepatocytes were plated in 96-well tissue culture plates and treated with APAP with or without TNFα and/or necrotic RNA. In separate wells, the cells were stimulated only with medium, TNFα or necrotic RNA (no APAP). After 24 h, Presto Blue was added to the cultures and incubated at 37°C, 5% CO_2_. After 1 h, the plates were read for a fluorescence signal that represents viable cells (*n* = 12 for each stimulus). ***p<0.001 when medium was compared with APAP; **p<0.01; when APAP alone or APAP+TNFα was compared with APAP+TNFα+necrotic RNA. #p<0.05 when necrotic RNA was compared with medium; ####p<0.0001 when TNFα was compared with medium.

Based on these findings and since the immunoneutralization of TLR3 in WT mice at 3 h after APAP significantly reduced levels of TNFα (**Figure 6A**), we hypothesized that TLR3 activation in the liver initially leads to TNFα production and the presence of this cytokine and TLR3 then contributes to APAP acute hepatotoxicity. We were able to confirm this hypothesis via *in vitro* experiments in which the viability of cultures of WT primary hepatocytes exposed to APAP+TNFα+necrotic RNA was significantly lower when compared to cultures of WT primary hepatocytes exposed to APAP alone, RNA from necrotic cells alone, or APAP+TNFα (**Figure 6B**). Interestingly, the addition of TNFα alone enhanced the viability of primary WT hepatocytes, confirming published findings that this cytokine has pleotropic effects on hepatocytes (**Figure 6B**). Together, these studies suggested that there was cooperation between TLR3- and TNFα-mediated activation events that had marked adverse effects on hepatocyte viability.

### TLR3 activation regulates JNK phosphorylation

**Figure 7:**
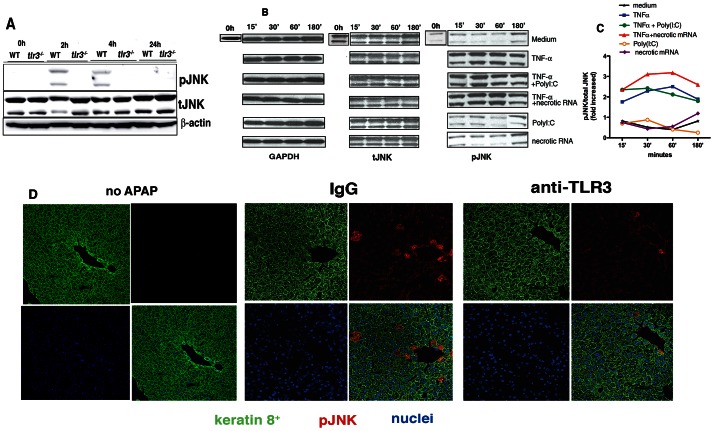
TLR3 regulates JNK activation. (**A**) Phosphorylation of JNK in liver samples was measured after APAP. Liver lysates of WT and *tlr3* ^−/−^ mice or (**B**) NmuLi were analyzed and the immunoblots shown are representative of three independent experiments while the immunoblots from 30′ time point are representative of four independent experiments. (**C**) The data in (**B**) were quantified by densitometry analysis using Image J Software. (**D**) Confocal analysis indicating the expression of p-JNK (red) and Keratin 8^+^ (green) in hepatocytes from WT mice that did not receive APAP (control), and that received either rabbit IgG or anti-TLR3 polyclonal antibody 3 h after APAP injection. DAPI stained the nuclei (blue). Tissues were collected 4 h post APAP injection. Representative images at 400*x* are shown (*n* = 5 per group).

Studies have demonstrated that JNK pathway activation contributes to APAP-induced liver hepatotoxicity [20], [21]. The effects of TLR3 deficiency on JNK activation in the liver after APAP challenge were next determined using Western blotting analysis. In WT mice, JNK phosphorylation peaked at 4 h after APAP, while activation of this kinase was undetected at 24 h (**Figure 7A**). In the absence of TLR3, the induction of JNK phosphorylation was not apparent at any of the time points analyzed (**Figure 7A**).

A previous study suggested that the inhibition of JNK might limit hepatotoxicity via reduced TNFα expression [21] and since, both TNFα and TLR3 are known activators of JNK kinases, studies were undertaken to further characterize the activation of this kinase in response to TLR3 activation, TNFαR activation or a combination of both. First, we checked the TLR3 expression on nMuli cells. Our results indicated that TNFα up-regulates the mRNA expression of TLR3 on these epithelial cells. Different from primary hepatocytes (**Figure 1D**), the presence of PolyI:C on the nMuli cells did not affect the expression of TLR3 (**Figure S7B**). Fasted nMuLi cells were exposed to TNFα alone, TLR3 ligands alone, or TNFα+TLR3 ligands. The combination of TNFα+necrotic RNA to cultured hepatocytes strongly induced JNK expression when compared with TNFα alone or TNFα+PolyI:C treatments but presence of necrotic RNA or PolyI:C alone did not result in p-JNK activation at the earlier time points analyzed (**Figure 7B–C**).

To confirm that the activation of TLR3 regulates the phosphorylation of JNK *in vivo*, the expression of p-JNK was analyzed by immunofluorescence in WT livers from mice treated with rabbit IgG or anti-TLR3 pAb at 4h after APAP. An immunofluorescence analysis did not reveal any positive cells for pJNK in untreated WT mice livers. In contrast, the numbers keratin 8+ pJNK+ cells and was lower in APAP-challenged WT mice that received anti-TLR3 pAb when compared with APAP-challenged WT mice that received control IgG (**Figure 7D**). Thus, these observations suggest that TNFα and TLR3 and cooperate in the activation of JNK in liver epithelial cells, indicating a potential mechanism via which TLR3 signaling amplifies APAP-induced liver injury.

## Discussion

The nature of the factors contributing to APAP-induced hepatotoxicity is not well understood, but tissue necrosis might provide a major ‘danger’ signal that contributes to further injury and cell death [31]. APAP overdose promotes the liver injury that involves the death of hepatocytes thereby leading to the release of several intracellular components including heat shock proteins (hsp), HMGB1, DNA, and RNA. Hsp and HMGB1 are agonists for TLR2 and TLR4 [32], and DNA can activate TLR9 while RNA is an endogenous ligand for TLR3 [33]. Although TLRs such as TLR9 and TLR4 activation contribute to APAP-induced liver toxicity [8], [34], the role of TLR3 in APAP hepatotoxicity is controversial [16]. In the present study a non-lethal dose of APAP induced TLR3 expression in WT mice, and this increased TLR3 expression in the liver and was localized to both immune (F4/80 and CD11c) cells and liver resident (hepatocytes and endothelial) cells. The absence of TLR3 in *tlr3* ^−/−^ mice had no discernable effect on liver expression of Cy2e1 (P450) nor did its absence appear to affect the levels of APAP in challenged mice. Studies employing *tlr3* ^−/−^ mice or neutralizing antibodies targeting TLR3 in APAP-challenged WT mice revealed that the absence of TLR3 activation significantly attenuated the extent of hepatotoxicity. In addition, *in vitro* studies with cultured primary hepatocytes or liver epithelial cells (i.e. NmuLi cells) demonstrated that TLR3 activation contributed to their injury/death. Specifically, TLR3 activation via recognition of endogenous RNA along with TNFα caused the death of cultured primary hepatocytes and robust activation of the JNK pathway in NmuLi cells exposed to APAP. TLR3 activation, particularly in the presence of TNFα during APAP challenge, appeared to be a key event in this acute liver injury setting. Our data also suggested that a signaling consequence of these combined stimuli was the phosphorylation of JNK.

Although it has been previously shown that TLR3 is expressed in cells of hematopoietic origin including DCs [35] and macrophages [36], more recent studies have shown that non-immune cells such as astrocytes [37], Schwann cells [14], glomerular mesangial cells [38], epithelial cells [39], fibroblasts [40], and keratinocytes [41] express this TLR in chronic and acute inflammation in the absence of virus or viral byproducts [11], [42] [10]. Interestingly, siRNA directly activates TLR3 on the cell surface of blood endothelial cells [43] suggesting that the regulation and localization of TLR3 are different in each cell type depending of the injury. Herein, following APAP challenge, it was apparent that TLR3 protein expression was localized to keratin 8^+^ hepatocytes (note that only hepatocytes express keratin 8), CD34+ cells, CD11c+ cells, and F4/80+ cells. Further analysis of primary hepatocytes using *in vitro* approaches revealed that the expression of TLR3 in primary hepatocytes was induced following exposure to a TLR3 ligand alone (PolyI:C) or a combination of APAP, TNFα, and PolyI:C. As to the importance of TLR3 expression by non-immune and immune cells during APAP-induced hepatotoxicity, BM chimeric experiments indicated that both cell families contributed to the liver injury induced by APAP challenge in mice.

*Tlr3^−/−^* mice exhibited significantly less liver injury and significantly less circulating AST and ALT levels at all time points examined after APAP challenge when compared with similarly challenged WT mice. Likewise *tlr3^−/−^* mice exhibited markedly less lung injury, which is a pathologic feature of APAP challenge in mice, previously described by Neff and colleagues [44]. Finally, *tlr3^−/−^* mice were resistant to a lethal dose of 500 mg/kg APAP. The diminished liver injury in APAP-challenged *tlr3^−/−^* mice did not appear to be a consequence of altered liver GSH levels or metabolism of APAP since the *tlr3^−/−^* and WT mice showed a similar loss in GSH, equivalent Cyp2e1 protein expression and identical serum APAP levels at various times after APAP challenge. The marked reduction of liver injury evident by histological and biochemical analysis in APAP-challenged *tlr3^−/−^* mice was duplicated in APAP-challenged WT mice treated with a neutralizing antibody directed against TLR3. Anti-TLR3 antibody approaches have been described in other injury models such as sepsis [11], gut ischemia [11], and lung hyperoxia [45]. In the present study, we observed that serum AST and ALT levels were significantly reduced and the histological appearance of the liver tissue was markedly improved following the administration of this antibody at 3 h after the induction APAP challenge. Although it is not presently clear how an antibody-based approach against this TLR is working, there are several examples in the literature clearly demonstrating that TLR3 expression is not restricted to the endosomal compartment and is in fact also localized at the cell membrane particularly during inflammatory conditions [35], [43] [11], [46]. Thus, the use of antibody directed against TLR3 might represent an attractive therapeutic approach for treatment of drug-induced acute liver failure.

The factors contributing to poorly regulated inflammatory responses such as those observed in APAP-induced hepatoxicity are not well understood, but tissue necrosis might provide a major ‘danger’ signal that inappropriately amplifies inflammation [31]. An earlier report by Lang *et al.* suggested that TLR3 activation triggered liver autoimmune disease via the induction of IFNα and TNFα [47]. In addition, Huys *et al.* demonstrated that type I IFNs represent pivotal mediators in TNFα-induced inflammatory shock, leading to generalized cell death [48]. In the present study, we noted that the absence of TLR3 blunted the generation of CC and CXC chemokine levels in whole liver at various times after APAP challenge. While these chemokines might have a role in the overall liver injury induced by APAP as some other studies have demonstrated [22] [44], [49], we hypothesized that TNFα was a more critical inflammatory cytokine in this model. Indeed, the pleiotropic biological effects of TNFα are well known in the liver and appear to be a consequence of its ability to initiate different intracellular signaling pathways [19], and consequently either contribute to liver regeneration or injury [6], [29], [50], [51]. Upon binding of the TNFα receptor, TRADD, TRAF2, and RIP are recruited forming complex I. The signaling from complex I leads to NF-κB activation and also the activation of p38, ERK, and JNK MAP Kinases [52]. Numerous studies have shown that the crosstalk between the NF-κB and JNK pathways determines the biological consequences of TNFα stimulation. Following APAP challenge, hepatocyte fate is controlled by the intensity and duration of JNK activation [19], and JNK activation via TNFα has a central role in the pathogenic consequences of this drug [21]. The binding of PAMPs to TLR3 leads to the activation of JNK and the transcription factor AP-1 [53]. Based on our findings, TNFα and TLR3 cooperate to contribute to APAP-induced injury in three possible situations: 1. The induction of necrosis leads to release of damage-associated molecular pattern molecules (DAMPs) (necrotic RNA) that stimulate TLR3 mainly on F480 and CD11c positive cells that results in TNFα secretion. The higher amounts of TNFα+necrotic RNA cooperate to increase the activation of JNK; 2. As in 1., the induction of necrosis by APAP leads to release of necrotic RNA that stimulates TLR3 in F480 and CD11c. However it is possible that the high levels of TNFα produced by these cells directly bind to hepatocytes independent of TLR3 leading to increase JNK; and 3. Both pathways can cooperate and amplify the liver injury post APAP.

In summary, *tlr3^−/−^* mice showed a marked attenuation in APAP-induced liver injury in the absence of a known TLR3 stimulus such as a viral challenge. Use of either a monoclonal or polyclonal neutralizing antibody directed against TLR3 recapitulated the findings observed in the gene deficient mice; histologic and biochemical evidence of liver injury was significantly reduced following the administration of this antibody after APAP challenge. Thus, this study provides both a target and putative mechanism for APAP-induced liver injury and suggests an effective strategy to limit APAP-induced acute liver failure.

## Supporting Information

Figure S1 **TLR3 protein expression is absent from** ***tlr3^−/−^*** **mice.** Confocal immunofluorescence analysis of (**A**) untreated WT livers showed basal expression of TLR3 (red). (**B**) Confocal immunofluorescence analysis of livers from *tlr3* ^−/−^ mice at 24 h after APAP injection is shown. No TLR3^+^ cells were apparent in the TLR3 gene deficient mice (red). Keratin 8^+^ hepatocytes appear green and DAPI^+^ nuclei appear blue. Shown are representative sections from groups of 5 mice. Magnification: ×200. (**C**) WT mice received: 1) saline vehicle; 2) N-acetyl-cysteine (NAC; used to treat patients with APAP overdose) (i.p. 100 mg/Kg) at 1 h after APAP (300 mg/Kg). The expression of TLR3 was analyzed 24 h post APAP challenge. Data are mean±SEM of two independent experiments. No statistical difference was found.
(TIF)

Figure S2 **C57BL/6 WT mice from Taconic and Jackson Laboratories presented similar APAP-induced liver injury.** Groups of fasted WT (*n* = 9 each group) and *tlr3* ^−/−^ mice received a single dose of APAP (300 mg/kg; i.p. injection), and serum ALT and AST were analyzed at 24 h after APAP challenge.
(TIF)

Figure S3 **TLR3-mediated APAP liver injury requires TLR3 signaling in both hematopoietic and non-hematopoietic cells.** Whole bone marrow (BM) cells were harvested from WT or *tlr3^−/−^* mice by flushing femurs and tibiae with PBS. Chimeric mice were generated by transferring donor BM cells into irradiated recipients using the following recipient/BM-donor combinations of WT and *tlr3^−/−^* mice: WT→WT BM (*n* = 5), WT→*tlr3−/−* BM (*n* = 5), *tlr3−/−*→WT BM (*n* = 5), and *tlr3−/−*→*tlr3−/−* BM (*n* = 3). The recipients were lethally irradiated (2 exposures of 900rads at 3 h apart). Twenty-four hours after irradiation, 10^6^ BM cells were injected i.v. into the recipients. The animals were allowed to recover for 12 weeks to ensure full reconstitution of hematopoietic cells. The mice were challenged with 300 mg/Kg of APAP. ALT, AST levels and histology were analyzed 24 h post APAP (Original magnification:×200). *p<0.05; **p<0.01, when all groups were compared with WT→WT BM chimeras (Tukey's multiple comparisons test).
(TIF)

Figure S4 **Immunoneutralization of TLR3 attenuated the APAP-induced hepatotoxicity in WT mice.** Groups of fasted WT mice were treated with either rabbit IgG or polyclonal anti-TLR3 antibody 3 h after an i.p. injection of 300 mg/kg of APAP injection, and analyzed 24 h later. (**A**) AST and ALT serum levels from WT mice that received either anti-TLR3 antibody or control IgG are indicated. (**B**) Representative liver sections stained with H&E or (**C**) PAS from WT mice that received either IgG or anti-TLR3 antibody at 3 h after APAP challenge. Magnification: 200×. (**D**) Lung sections of APAP-challenged WT mice that received anti-TLR3 antibody exhibited less epithelial injury whereas pronounced pulmonary epithelial damage was apparent in WT mice that received IgG. Magnification: ×400. Data in all panels are representative of two independent experiments (*n* = 5 mice per group). *p<0.05; when rabbit IgG are compared with anti-TLR3 antibody-treated mice.
(TIF)

Figure S5 **Immunoneutralization of TLR3 after APAP markedly reduced inflammatory chemokines levels in liver.** Groups of fasted WT mice received either rabbit IgG or anti-TLR3 3 h after APAP injection (300 mg/kg; i.p. injection), and analyzed at 24 h after APAP challenge. CC chemokines (CCL2, CCL3, and CCL5) and CXC chemokines (KC and CXCL10) protein levels were measured in livers homogenates using either Bioplex or ELISA techniques. *p<0.05; when rabbit IgG were compared with anti-TLR3 antibody-treated mice.
(TIF)

Figure S6 **Type I IFN activation does not regulates liver injury induced by APAP.** Groups of fasted WT and knockouts mice received an i.p. injection of 300 mg/kg of APAP (**A**) Transcript levels of IFNα4 and IFNß1 in whole liver homogenates from WT and *tlr3* ^−/−^ mice at 24 h post APAP. (**B**) TNFα and (**C**) chemokines levels was measured in liver homogenates from WT and *IFN*α*R* ^−/−^ mice by ELISA and Bioplex, respectively. (**D**) Serum ALT and AST levels in WT and *IFN*α*R* ^−/−^ mice were similar at 24 h after APAP challenge. (**E**) Areas of hepatic injury and hepatocyte death were similar in both groups of mice at 24 h after APAP (H&E staining) and (**F**) PAS staining (Original magnification: ×200). Data are representative of *n* = 5 for WT and *n* = 7 for *IFN*α*R* ^−/−^ mice.
(TIF)

Figure S7 (**A**) **Profile of TNFα staining in liver cells.** Histograms represent the three distinct cell populations expressing TNFα. Gray histogram represents Isotype control; green histogram: CD11c^+^F4/80^−^; blue histogram: CD11c^−^F4/80^+^; orange histogram: CD11c^+^F4/80^+^ cells. (**B**) **Expression of TLR3 transcript levels in nMuli cells.** 5×10^5^ cells/well were plated in 24 well-plates and stimulated with the indicated stimulus (10 mM of APAP, 20 ng/ml of TNFα, 10 µg/ml of PolyI:C) for 24 h. Bars represents mean ± SEM of two independent experiments. ***p<0.0001 when APAP is compared with medium; **p<0.01, when APAP+TNFα is compared with APAP; #p<0.05, when TNFα is compared with medium.
(TIF)

## References

[1] Larson AM, Polson J, Fontana RJ, Davern TJ, Lalani E, et al (2005) Acetaminophen-induced acute liver failure: results of a United States multicenter, prospective study. Hepatology 42: 1364–1372. 16317692 10.1002/hep.20948

[2] Lee WM (2007) Acetaminophen toxicity: changing perceptions on a social/medical issue. Hepatology 46: 966–970. 17894320 10.1002/hep.21926

[3] Kaplowitz N (2004) Acetaminophen hepatoxicity: what do we know, what don't we know, and what do we do next? Hepatology 40: 23–26. 15239082 10.1002/hep.20312

[4] Liu ZX, Han D, Gunawan B, Kaplowitz N (2006) Neutrophil depletion protects against murine acetaminophen hepatotoxicity. Hepatology 43: 1220–1230. 16729305 10.1002/hep.21175

[5] Liu ZX, Govindarajan S, Kaplowitz N (2004) Innate immune system plays a critical role in determining the progression and severity of acetaminophen hepatotoxicity. Gastroenterology 127: 1760–1774. 15578514 10.1053/j.gastro.2004.08.053

[6] Blazka ME, Wilmer JL, Holladay SD, Wilson RE, Luster MI (1995) Role of proinflammatory cytokines in acetaminophen hepatotoxicity. Toxicol Appl Pharmacol 133: 43–52. 7597709 10.1006/taap.1995.1125

[7] Marshak-Rothstein A (2006) Toll-like receptors in systemic autoimmune disease. Nat Rev Immunol 6: 823–835. 17063184 10.1038/nri1957 PMC7097510

[8] Imaeda AB, Watanabe A, Sohail MA, Mahmood S, Mohamadnejad M, et al (2009) Acetaminophen-induced hepatotoxicity in mice is dependent on Tlr9 and the Nalp3 inflammasome. J Clin Invest 119: 305–314. 19164858 10.1172/JCI35958 PMC2631294

[9] McGill MR, Sharpe MR, Williams CD, Taha M, Curry SC, et al (2012) The mechanism underlying acetaminophen-induced hepatotoxicity in humans and mice involves mitochondrial damage and nuclear DNA fragmentation. J Clin Invest 122: 1574–1583. 22378043 10.1172/JCI59755 PMC3314460

[10] Brentano F, Schorr O, Gay RE, Gay S, Kyburz D (2005) RNA released from necrotic synovial fluid cells activates rheumatoid arthritis synovial fibroblasts via Toll-like receptor 3. Arthritis Rheum 52: 2656–2665. 16142732 10.1002/art.21273

[11] Cavassani KA, Ishii M, Wen H, Schaller MA, Lincoln PM, et al (2008) TLR3 is an endogenous sensor of tissue necrosis during acute inflammatory events. J Exp Med 205: 2609–2621. 18838547 10.1084/jem.20081370 PMC2571935

[12] Lai Y, Di Nardo A, Nakatsuji T, Leichtle A, Yang Y, et al (2009) Commensal bacteria regulate Toll-like receptor 3-dependent inflammation after skin injury. Nat Med 15: 1377–1382. 19966777 10.1038/nm.2062 PMC2880863

[13] Bernard JJ, Cowing-Zitron C, Nakatsuji T, Muehleisen B, Muto J, et al (2012) Ultraviolet radiation damages self noncoding RNA and is detected by TLR3. Nat Med 10.1038/nm.2861 PMC3812946 22772463

[14] Lee H, Jo EK, Choi SY, Oh SB, Park K, et al (2006) Necrotic neuronal cells induce inflammatory Schwann cell activation via TLR2 and TLR3: implication in Wallerian degeneration. Biochem Biophys Res Commun 350: 742–747. 17027917 10.1016/j.bbrc.2006.09.108

[15] Yin S, Gao B (2010) Toll-like receptor 3 in liver diseases. Gastroenterol Res Pract 2010 10.1155/2010/750904 PMC2948910 20936107

[16] Ghaffari AA, Chow EK, Iyer SS, Deng JC, Cheng G (2011) Polyinosinic-polycytidylic acid suppresses acetaminophen-induced hepatotoxicity independent of type I interferons and toll-like receptor 3. Hepatology 53: 2042–2052. 21433044 10.1002/hep.24316 PMC3103596

[17] Schwabe RF, Brenner DA (2006) Mechanisms of Liver Injury. I. TNF-alpha-induced liver injury: role of IKK, JNK, and ROS pathways. Am J Physiol Gastrointest Liver Physiol 290: G583–589. 16537970 10.1152/ajpgi.00422.2005

[18] Swantek JL, Cobb MH, Geppert TD (1997) Jun N-terminal kinase/stress-activated protein kinase (JNK/SAPK) is required for lipopolysaccharide stimulation of tumor necrosis factor alpha (TNF-alpha) translation: glucocorticoids inhibit TNF-alpha translation by blocking JNK/SAPK. Mol Cell Biol 17: 6274–6282. 9343388 10.1128/mcb.17.11.6274 PMC232478

[19] Papa S, Bubici C, Zazzeroni F, Franzoso G (2009) Mechanisms of liver disease: cross-talk between the NF-kappaB and JNK pathways. Biol Chem 390: 965–976. 19642868 10.1515/BC.2009.111 PMC2775491

[20] Gunawan BK, Liu ZX, Han D, Hanawa N, Gaarde WA, et al (2006) c-Jun N-terminal kinase plays a major role in murine acetaminophen hepatotoxicity. Gastroenterology 131: 165–178. 16831600 10.1053/j.gastro.2006.03.045

[21] Henderson NC, Pollock KJ, Frew J, Mackinnon AC, Flavell RA, et al (2007) Critical role of c-jun (NH2) terminal kinase in paracetamol- induced acute liver failure. Gut 56: 982–990. 17185352 10.1136/gut.2006.104372 PMC1994347

[22] Hogaboam CM, Bone-Larson CL, Steinhauser ML, Matsukawa A, Gosling J, et al (2000) Exaggerated hepatic injury due to acetaminophen challenge in mice lacking C-C chemokine receptor 2. Am J Pathol 156: 1245–1252. 10751350 10.1016/S0002-9440(10)64995-4 PMC1876888

[23] Hu B, Colletti LM (2008) Stem cell factor and c-kit are involved in hepatic recovery after acetaminophen-induced liver injury in mice. Am J Physiol Gastrointest Liver Physiol 295: G45–G53. 18467506 10.1152/ajpgi.00024.2008 PMC2494727

[24] Zellweger RM, Prestwood TR, Shresta S (2010) Enhanced infection of liver sinusoidal endothelial cells in a mouse model of antibody-induced severe dengue disease. Cell Host Microbe 7: 128–139. 20153282 10.1016/j.chom.2010.01.004 PMC2824513

[25] Mencin A, Kluwe J, Schwabe RF (2009) Toll-like receptors as targets in chronic liver diseases. Gut 58: 704–720. 19359436 10.1136/gut.2008.156307 PMC2791673

[26] Matsumura T, Ito A, Takii T, Hayashi H, Onozaki K (2000) Endotoxin and cytokine regulation of toll-like receptor (TLR) 2 and TLR4 gene expression in murine liver and hepatocytes. J Interferon Cytokine Res 20: 915–921. 11054280 10.1089/10799900050163299

[27] Li K, Chen Z, Kato N, Gale M Jr, Lemon SM (2005) Distinct poly(I-C) and virus-activated signaling pathways leading to interferon-beta production in hepatocytes. J Biol Chem 280: 16739–16747. 15737993 10.1074/jbc.M414139200

[28] Bourdi M, Davies JS, Pohl LR (2011) Mispairing C57BL/6 substrains of genetically engineered mice and wild-type controls can lead to confounding results as it did in studies of JNK2 in acetaminophen and concanavalin A liver injury. Chem Res Toxicol 24: 794–796. 21557537 10.1021/tx200143x PMC3157912

[29] Blazka ME, Bruccoleri A, Simeonova PP, Germolec DR, Pennypacker KR, et al (1996) Acetaminophen-induced hepatotoxicity is associated with early changes in AP-1 DNA binding activity. Res Commun Mol Pathol Pharmacol 92: 259–273. 8827825

[30] Czaja MJ (2003) The future of GI and liver research: editorial perspectives. III. JNK/AP-1 regulation of hepatocyte death. Am J Physiol Gastrointest Liver Physiol 284: G875–879. 12736142 10.1152/ajpgi.00549.2002

[31] Matzinger P (1994) Tolerance, danger, and the extended family. Annu Rev Immunol 12: 991–1045. 8011301 10.1146/annurev.iy.12.040194.005015

[32] Tsan MF, Gao B (2004) Endogenous ligands of Toll-like receptors. J Leukoc Biol 76: 514–519. 15178705 10.1189/jlb.0304127

[33] Kariko K, Ni H, Capodici J, Lamphier M, Weissman D (2004) mRNA is an endogenous ligand for Toll-like receptor 3. J Biol Chem 279: 12542–12550. 14729660 10.1074/jbc.M310175200

[34] Yohe HC, O'Hara KA, Hunt JA, Kitzmiller TJ, Wood SG, et al (2006) Involvement of Toll-like receptor 4 in acetaminophen hepatotoxicity. Am J Physiol Gastrointest Liver Physiol 290: G1269–1279. 16439473 10.1152/ajpgi.00239.2005

[35] Matsumoto M, Funami K, Tanabe M, Oshiumi H, Shingai M, et al (2003) Subcellular localization of Toll-like receptor 3 in human dendritic cells. J Immunol 171: 3154–3162. 12960343 10.4049/jimmunol.171.6.3154

[36] Jiang W, Sun R, Wei H, Tian Z (2005) Toll-like receptor 3 ligand attenuates LPS-induced liver injury by down-regulation of toll-like receptor 4 expression on macrophages. Proc Natl Acad Sci U S A 102: 17077–17082. 16287979 10.1073/pnas.0504570102 PMC1287976

[37] Farina C, Krumbholz M, Giese T, Hartmann G, Aloisi F, et al (2005) Preferential expression and function of Toll-like receptor 3 in human astrocytes. J Neuroimmunol 159: 12–19. 15652398 10.1016/j.jneuroim.2004.09.009

[38] Patole PS, Grone HJ, Segerer S, Ciubar R, Belemezova E, et al (2005) Viral double-stranded RNA aggravates lupus nephritis through Toll-like receptor 3 on glomerular mesangial cells and antigen-presenting cells. J Am Soc Nephrol 16: 1326–1338. 15772251 10.1681/ASN.2004100820

[39] Sha Q, Truong-Tran AQ, Plitt JR, Beck LA, Schleimer RP (2004) Activation of airway epithelial cells by toll-like receptor agonists. Am J Respir Cell Mol Biol 31: 358–364. 15191912 10.1165/rcmb.2003-0388OC

[40] Rudd BD, Burstein E, Duckett CS, Li X, Lukacs NW (2005) Differential role for TLR3 in respiratory syncytial virus-induced chemokine expression. J Virol 79: 3350–3357. 15731229 10.1128/JVI.79.6.3350-3357.2005 PMC1075725

[41] Begon E, Michel L, Flageul B, Beaudoin I, Jean-Louis F, et al (2007) Expression, subcellular localization and cytokinic modulation of Toll-like receptors (TLRs) in normal human keratinocytes: TLR2 up-regulation in psoriatic skin. Eur J Dermatol 17: 497–506. 17951129 10.1684/ejd.2007.0264

[42] Patole PS, Pawar RD, Lech M, Zecher D, Schmidt H, et al (2006) Expression and regulation of Toll-like receptors in lupus-like immune complex glomerulonephritis of MRL-Fas(lpr) mice. Nephrol Dial Transplant 21: 3062–3073. 16954173 10.1093/ndt/gfl336

[43] Cho WG, Albuquerque RJ, Kleinman ME, Tarallo V, Greco A, et al (2009) Small interfering RNA-induced TLR3 activation inhibits blood and lymphatic vessel growth. Proc Natl Acad Sci U S A 106: 7137–7142. 19359485 10.1073/pnas.0812317106 PMC2678451

[44] Neff SB, Neff TA, Kunkel SL, Hogaboam CM (2003) Alterations in cytokine/chemokine expression during organ-to-organ communication established via acetaminophen-induced toxicity. Exp Mol Pathol 75: 187–193. 14611809 10.1016/s0014-4800(03)00096-0

[45] Murray LA, Knight DA, McAlonan L, Argentieri R, Joshi A, et al (2008) Deleterious role of TLR3 during hyperoxia-induced acute lung injury. Am J Respir Crit Care Med 178: 1227–1237. 18849495 10.1164/rccm.200807-1020OC

[46] Kleinman ME, Yamada K, Takeda A, Chandrasekaran V, Nozaki M, et al (2008) Sequence- and target-independent angiogenesis suppression by siRNA via TLR3. Nature 452: 591–597. 18368052 10.1038/nature06765 PMC2642938

[47] Lang KS, Georgiev P, Recher M, Navarini AA, Bergthaler A, et al (2006) Immunoprivileged status of the liver is controlled by Toll-like receptor 3 signaling. J Clin Invest 116: 2456–2463. 16955143 10.1172/JCI28349 PMC1555644

[48] Huys L, Van Hauwermeiren F, Dejager L, Dejonckheere E, Lienenklaus S, et al (2009) Type I interferon drives tumor necrosis factor-induced lethal shock. J Exp Med 206: 1873–1882. 19687227 10.1084/jem.20090213 PMC2737160

[49] Bone-Larson CL, Hogaboam CM, Evanhoff H, Strieter RM, Kunkel SL (2001) IFN-gamma-inducible protein-10 (CXCL10) is hepatoprotective during acute liver injury through the induction of CXCR2 on hepatocytes. J Immunol 167: 7077–7083. 11739529 10.4049/jimmunol.167.12.7077

[50] Simpson KJ, Lukacs NW, McGregor AH, Harrison DJ, Strieter RM, et al (2000) Inhibition of tumour necrosis factor alpha does not prevent experimental paracetamol-induced hepatic necrosis. J Pathol 190: 489–494. 10700000 10.1002/(SICI)1096-9896(200003)190:4<489::AID-PATH534>3.0.CO;2-V

[51] Ishida Y, Kondo T, Tsuneyama K, Lu P, Takayasu T, et al (2004) The pathogenic roles of tumor necrosis factor receptor p55 in acetaminophen-induced liver injury in mice. J Leukoc Biol 75: 59–67. 14557383 10.1189/jlb.0403152

[52] Wullaert A, Heyninck K, Beyaert R (2006) Mechanisms of crosstalk between TNF-induced NF-kappaB and JNK activation in hepatocytes. Biochem Pharmacol 72: 1090–1101. 16934229 10.1016/j.bcp.2006.07.003

[53] Dahlberg JE, Lund E (2007) Micromanagement during the innate immune response. Sci STKE 2007: pe25. 17519424 10.1126/stke.3872007pe25

