# Supplementary figures and images for: Toll Like Receptor 3 Plays a Critical Role in the Progression and Severity of Acetaminophen-Induced Hepatotoxicity
Source: PLoS One. 2013 Jun 7;8(6):e65899. doi: 10.1371/journal.pone.0065899 (PMC3676358; doi:10.1371/journal.pone.0065899)

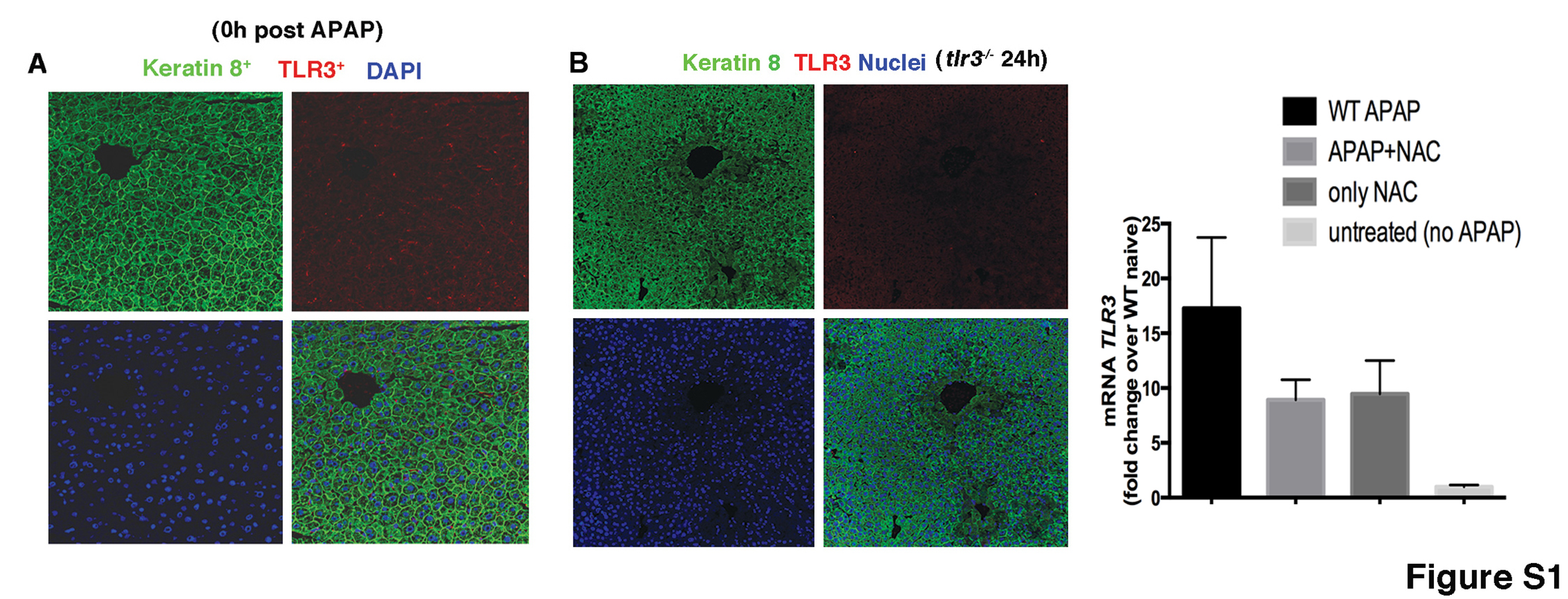

Supplement: Figure S1 — TLR3 protein expression is absent from tlr3−/− mice. Confocal immunofluorescence analysis of (A) untreated WT livers showed basal expression of TLR3 (red). (B) Confocal immunofluorescence analysis of livers from tlr3 −/− mice at 24 h after APAP injection is shown. No TLR3+ cells were apparent in the TLR3 gene deficient mice (red). Keratin 8+ hepatocytes appear green and DAPI+ nuclei appear blue. Shown are representative sections from groups of 5 mice. Magnification: ×200. (C) WT mice received: 1) saline vehicle; 2) N-acetyl-cysteine (NAC; used to treat patients with APAP overdose) (i.p. 100 mg/Kg) at 1 h after APAP (300 mg/Kg). The expression of TLR3 was analyzed 24 h post APAP challenge. Data are mean±SEM of two independent experiments. No statistical difference was found. (TIF) [file pone.0065899.s001.tif]

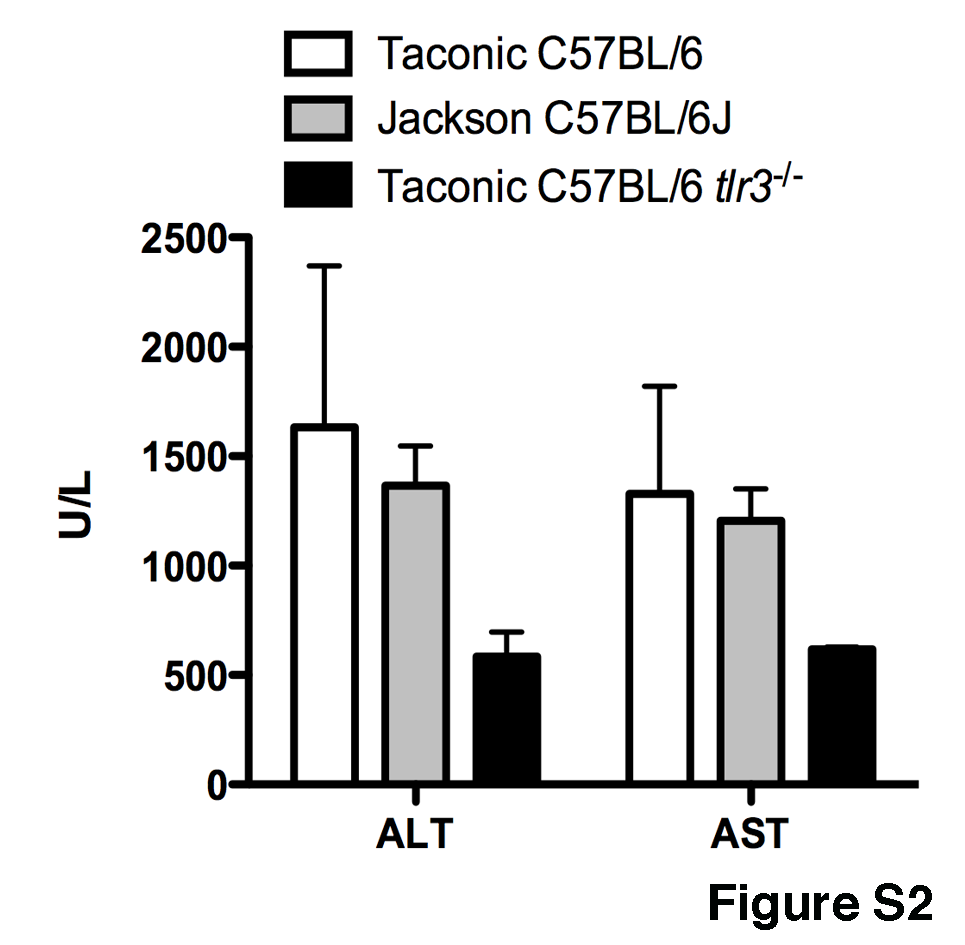

Supplement: Figure S2 — C57BL/6 WT mice from Taconic and Jackson Laboratories presented similar APAP-induced liver injury. Groups of fasted WT (n = 9 each group) and tlr3 −/− mice received a single dose of APAP (300 mg/kg; i.p. injection), and serum ALT and AST were analyzed at 24 h after APAP challenge. (TIF) [file pone.0065899.s002.tif]

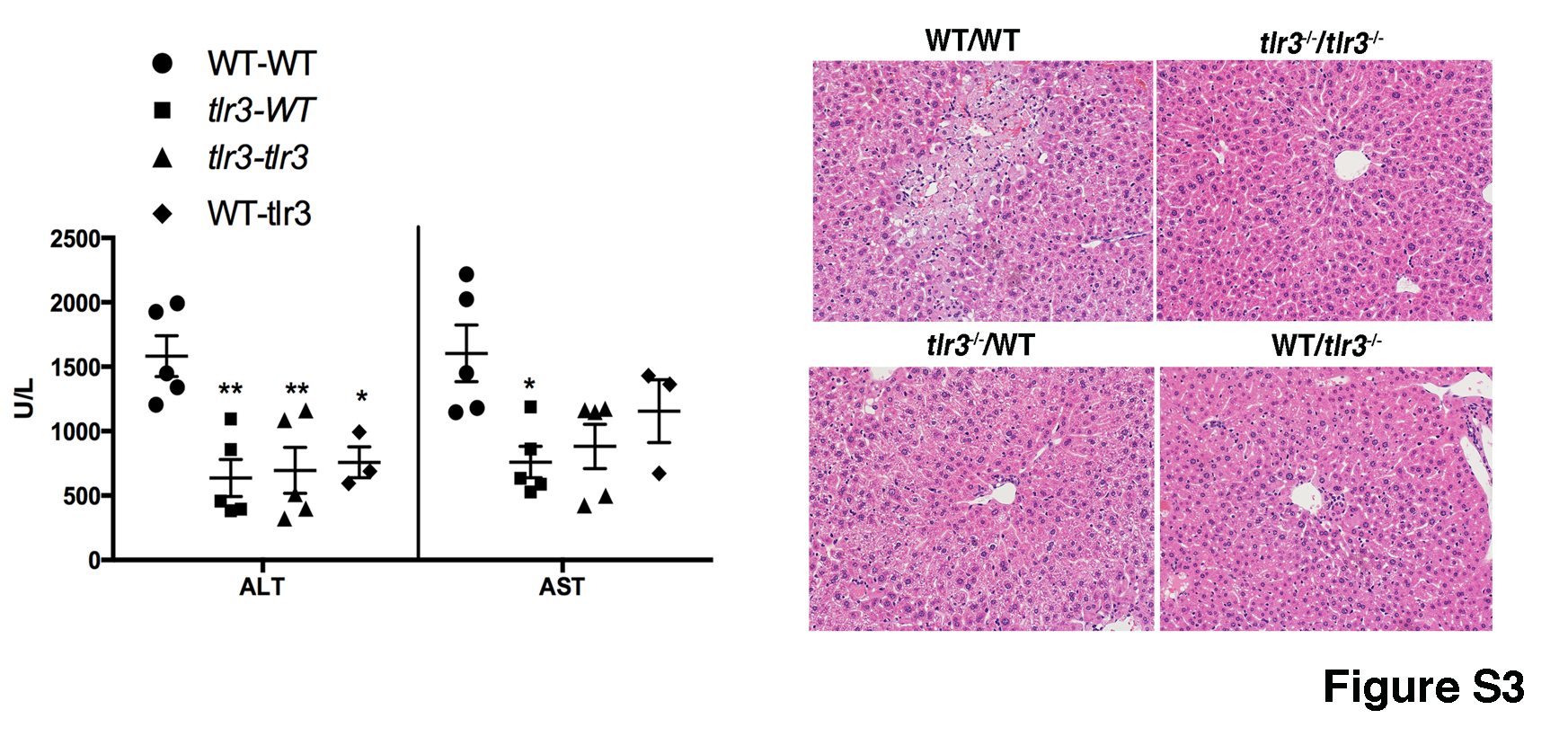

Supplement: Figure S3 — TLR3-mediated APAP liver injury requires TLR3 signaling in both hematopoietic and non-hematopoietic cells. Whole bone marrow (BM) cells were harvested from WT or tlr3−/− mice by flushing femurs and tibiae with PBS. Chimeric mice were generated by transferring donor BM cells into irradiated recipients using the following recipient/BM-donor combinations of WT and tlr3−/− mice: WT→WT BM (n = 5), WT→tlr3−/− BM (n = 5), tlr3−/−→WT BM (n = 5), and tlr3−/−→tlr3−/− BM (n = 3). The recipients were lethally irradiated (2 exposures of 900rads at 3 h apart). Twenty-four hours after irradiation, 106 BM cells were injected i.v. into the recipients. The animals were allowed to recover for 12 weeks to ensure full reconstitution of hematopoietic cells. The mice were challenged with 300 mg/Kg of APAP. ALT, AST levels and histology were analyzed 24 h post APAP (Original magnification:×200). *p<0.05; **p<0.01, when all groups were compared with WT→WT BM chimeras (Tukey's multiple comparisons test). (TIF) [file pone.0065899.s003.tif]

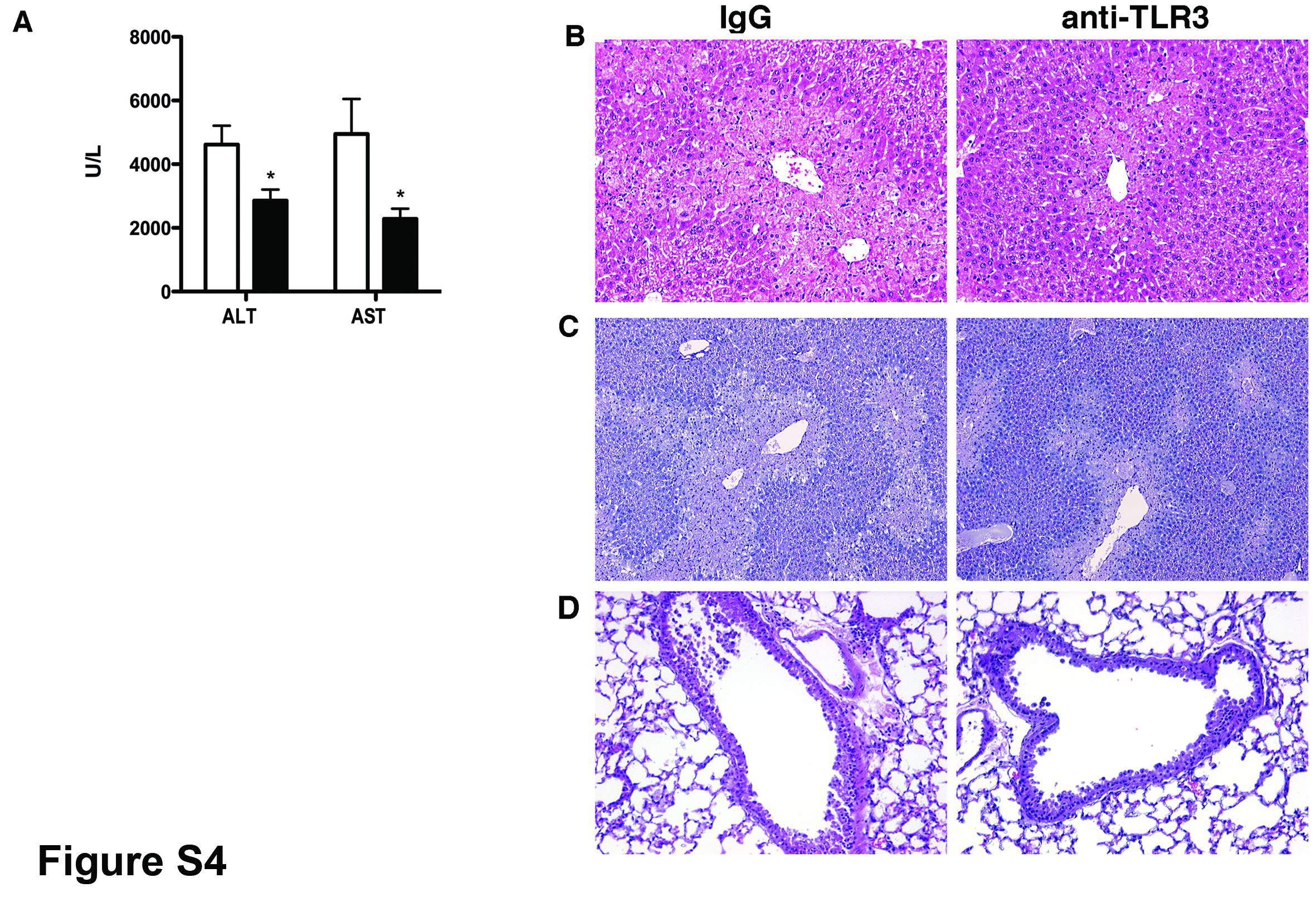

Supplement: Figure S4 — Immunoneutralization of TLR3 attenuated the APAP-induced hepatotoxicity in WT mice. Groups of fasted WT mice were treated with either rabbit IgG or polyclonal anti-TLR3 antibody 3 h after an i.p. injection of 300 mg/kg of APAP injection, and analyzed 24 h later. (A) AST and ALT serum levels from WT mice that received either anti-TLR3 antibody or control IgG are indicated. (B) Representative liver sections stained with H&E or (C) PAS from WT mice that received either IgG or anti-TLR3 antibody at 3 h after APAP challenge. Magnification: 200×. (D) Lung sections of APAP-challenged WT mice that received anti-TLR3 antibody exhibited less epithelial injury whereas pronounced pulmonary epithelial damage was apparent in WT mice that received IgG. Magnification: ×400. Data in all panels are representative of two independent experiments (n = 5 mice per group). *p<0.05; when rabbit IgG are compared with anti-TLR3 antibody-treated mice. (TIF) [file pone.0065899.s004.tif]

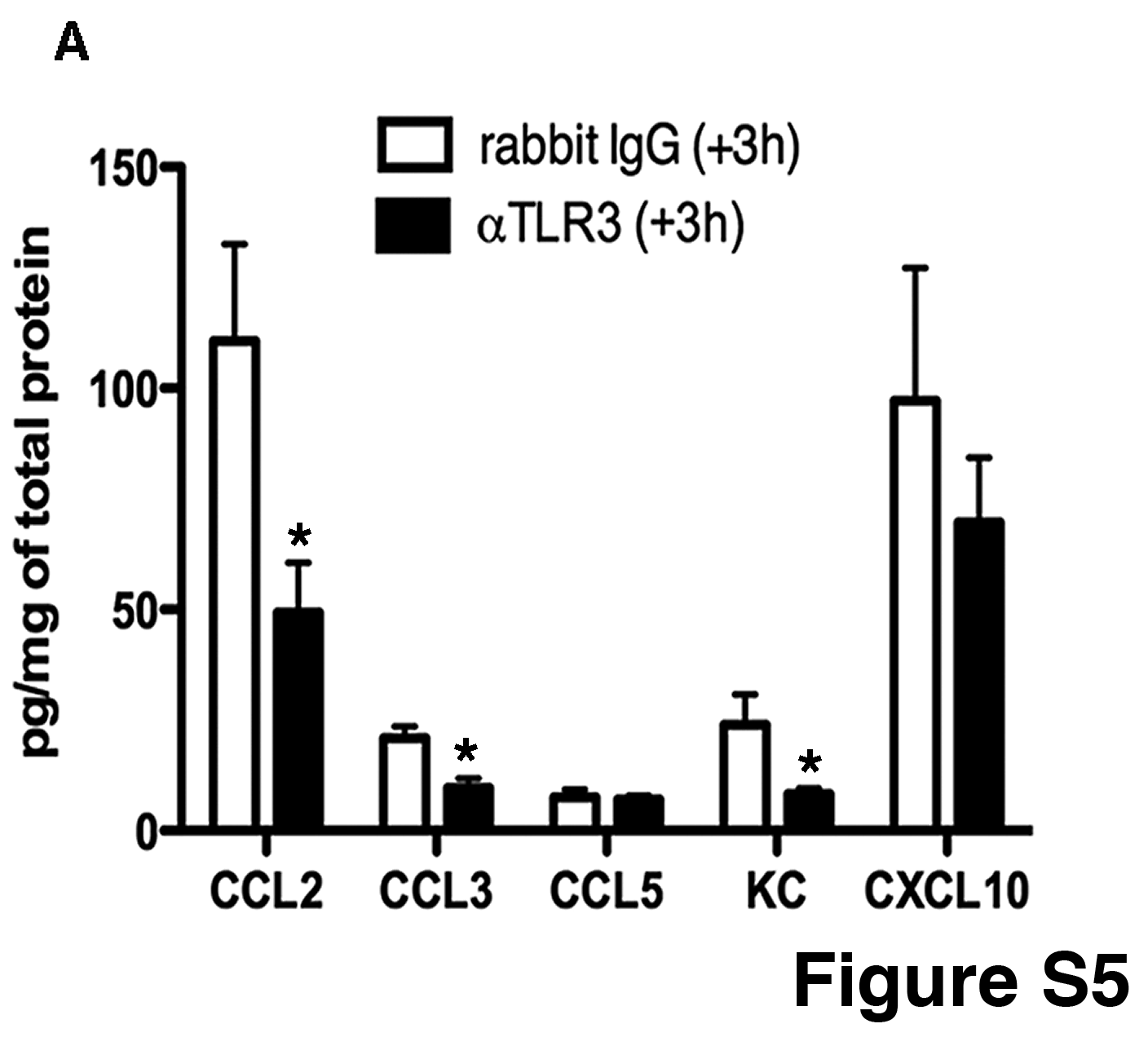

Supplement: Figure S5 — Immunoneutralization of TLR3 after APAP markedly reduced inflammatory chemokines levels in liver. Groups of fasted WT mice received either rabbit IgG or anti-TLR3 3 h after APAP injection (300 mg/kg; i.p. injection), and analyzed at 24 h after APAP challenge. CC chemokines (CCL2, CCL3, and CCL5) and CXC chemokines (KC and CXCL10) protein levels were measured in livers homogenates using either Bioplex or ELISA techniques. *p<0.05; when rabbit IgG were compared with anti-TLR3 antibody-treated mice. (TIF) [file pone.0065899.s005.tif]

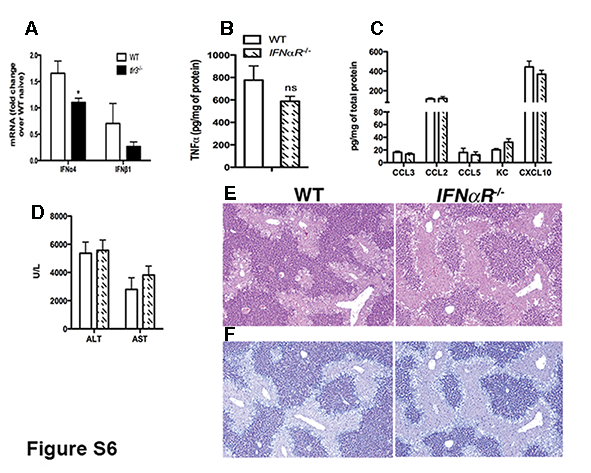

Supplement: Figure S6 — Type I IFN activation does not regulates liver injury induced by APAP. Groups of fasted WT and knockouts mice received an i.p. injection of 300 mg/kg of APAP (A) Transcript levels of IFNα4 and IFNß1 in whole liver homogenates from WT and tlr3 −/− mice at 24 h post APAP. (B) TNFα and (C) chemokines levels was measured in liver homogenates from WT and IFNαR −/− mice by ELISA and Bioplex, respectively. (D) Serum ALT and AST levels in WT and IFNαR −/− mice were similar at 24 h after APAP challenge. (E) Areas of hepatic injury and hepatocyte death were similar in both groups of mice at 24 h after APAP (H&E staining) and (F) PAS staining (Original magnification: ×200). Data are representative of n = 5 for WT and n = 7 for IFNαR −/− mice. (TIF) [file pone.0065899.s006.tif]

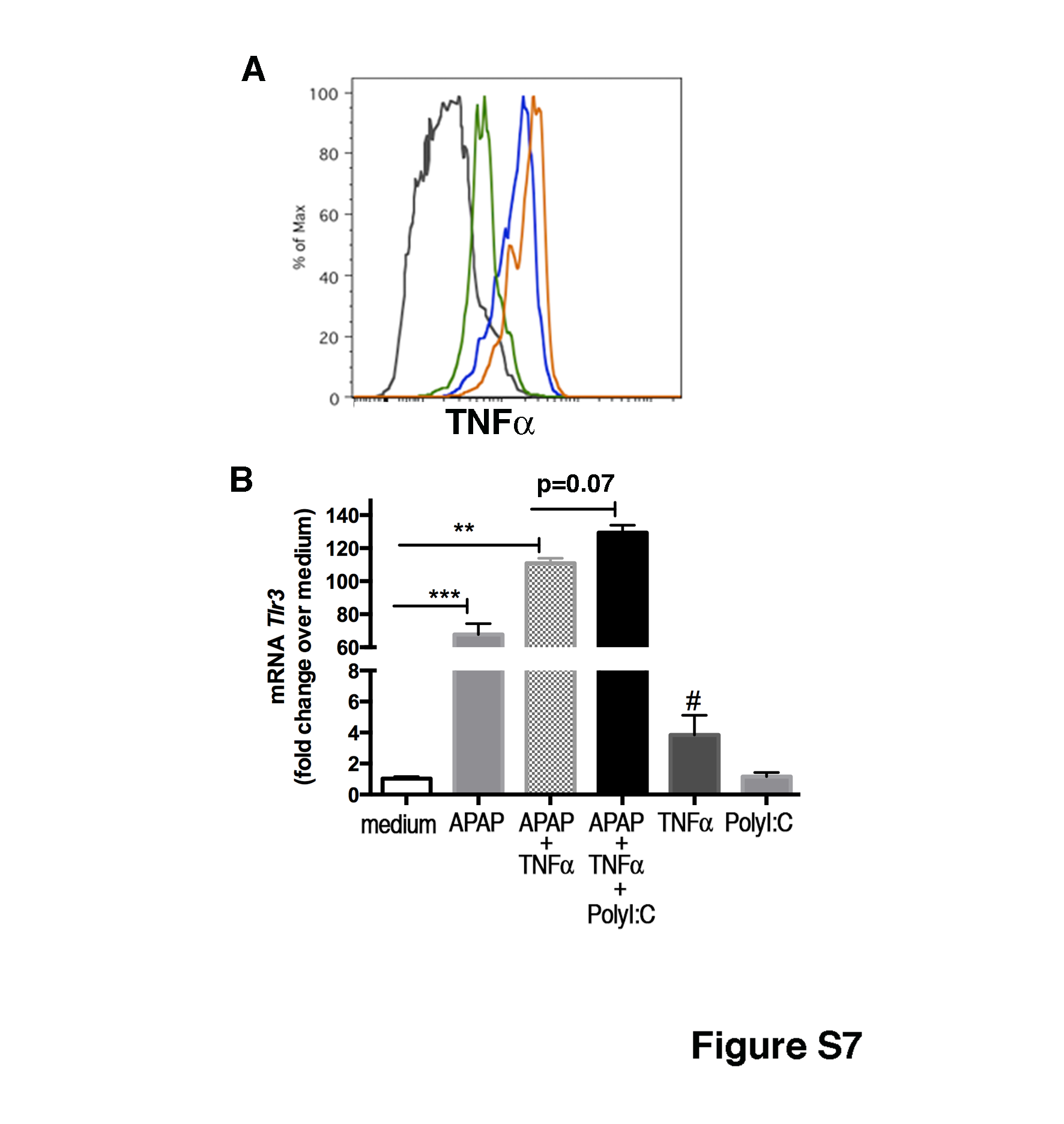

Supplement: Figure S7 — (A) Profile of TNFα staining in liver cells. Histograms represent the three distinct cell populations expressing TNFα. Gray histogram represents Isotype control; green histogram: CD11c+F4/80−; blue histogram: CD11c−F4/80+; orange histogram: CD11c+F4/80+ cells. (B) Expression of TLR3 transcript levels in nMuli cells. 5×10^5^ cells/well were plated in 24 well-plates and stimulated with the indicated stimulus (10 mM of APAP, 20 ng/ml of TNFα, 10 µg/ml of PolyI:C) for 24 h. Bars represents mean ± SEM of two independent experiments. ***p<0.0001 when APAP is compared with medium; **p<0.01, when APAP+TNFα is compared with APAP; #p<0.05, when TNFα is compared with medium. (TIF) [file pone.0065899.s007.tif]
